# Learning supervised embeddings for large scale sequence comparisons

**DOI:** 10.1371/journal.pone.0216636

**Published:** 2020-03-13

**Authors:** Dhananjay Kimothi, Pravesh Biyani, James M. Hogan, Akshay Soni, Wayne Kelly

**Affiliations:** 1 Department of ECE, Indraprastha Institute of Information Technology-Delhi, New Delhi, India; 2 School of Computer Science, Queensland University of Technology, Brisbane, Queensland, Australia; 3 Microsoft, Sunnyvale, California, United States of America; University of Cyprus, CYPRUS

## Abstract

Similarity-based search of sequence collections is a core task in bioinformatics, one dominated for most of the genomic era by exact and heuristic alignment-based algorithms. However, even efficient heuristics such as BLAST may not scale to the data sets now emerging, motivating a range of alignment-free alternatives exploiting the underlying lexical structure of each sequence. In this paper, we introduce two supervised approaches—SuperVec and SuperVecX—to learn sequence embeddings. These methods extend earlier Representation Learning (RepL) based methods to include class-related information for each sequence during training. Including class information ensures that related sequence fragments have proximal representations in the target space, better reflecting the structure of the domain. We show the quality of the embeddings learned through these methods on (i) sequence retrieval and (ii) classification tasks. We also propose an hierarchical tree-based approach specifically designed for the sequence retrieval problem. The resulting methods, which we term H-SuperVec or H-SuperVecX, according to their respective use of SuperVec or SuperVecX, learn embeddings across a range of feature spaces based on exclusive and exhaustive subsets of the class labels. Experiments show that the proposed methods perform better for retrieval and classification tasks over existing (unsupervised) RepL-based approaches. Further, the new methods are an order of magnitude faster than BLAST for the database retrieval task, supporting hybrid approaches that rapidly filter the collection so that only potentially relevant records remain. Such filtering of the original database allows slower but more accurate methods to be executed quickly over a far smaller dataset. Thus, we may achieve faster query processing and higher precision than before.

## 1 Introduction

Rapid comparison of molecular sequences is an essential task in bioinformatics, with applications including homology detection, annotation, and phylogenetic analysis [1]. For most of the genomic era, sequence comparison has relied primarily on a succession of exact and subsequently heuristic algorithms for sequence alignment, the most successful being BLAST, the *Basic Local Alignment Search Tool*, which has in various guises dominated the field since its introduction in 1990 [2]. Exact algorithms for finding global and local sequence alignments were introduced respectively in [3] and [4]. Here the alignment score constitutes a pseudo-metric and a principled yet time-consuming method for quantifying sequence similarity and divergence. A high alignment score is taken to indicate a high probability that the sequences are similar. However, this intuition may break down for isolated local alignments or more remote homology, and fail altogether under structural re-arrangement.

BLAST rapidly identifies short, high-scoring seed matches between the sequences, extending and combining these as long as a strong match score is maintained. As is well known, the approximate alignment and its significance are estimated using Karlin-Altschul statistics, and these are used to rank the results returned when queries are submitted to sequence collections. BLAST and more recent sequence searching methods such as MMseqs2 [5] uses word-based heuristics to avoid the computational penalties inherent in the dynamic programming based exact alignment algorithms which are quadratic in the sequence length —the complexity of aligning and hence computing the similarity of two sequences of length *n* is $O(n^{2})$. Yet the exponential increase in the availability of sequence data as a result of successive ‘next-generation sequencing’ (NGS) technologies has highlighted the limitations even of successful heuristics of this kind, motivating research into comparison methods that do not rely on the underlying primitive of sequence alignment. Such alignment-free methods construct similarity through alternative features, usually based upon the bag-of-words (BOW) representation familiar from information retrieval, and have the additional virtue of robustness in the presence of structural re-arrangements.

The construction of BOW for sequences follows two steps: (i) splitting the biological sequence into *k-mers* (or words), and (ii) computing the statistics for either an individual *k-mer* or a *k-mer* set. The limitations of the BOW representation lie in its high dimensionality and inability to capture patterns inherent in the sequence, patterns that might otherwise support more meaningful comparison. Recent advances in learning distributed representations for text processing have led to techniques that provide a semantically meaningful, dense vector representation of words present in a large corpus of documents. Such Representation learning (RepL) approaches in text processing rely on the distributional hypothesis of semantics, the idea that words which frequently co-occur support a common meaning. The best known of these approaches is Word2Vec, due to Mikolov et.al [6]. These techniques may also be applied to biological sequences to analyse, compare, and perform downstream machine learning tasks for various applications. Initial research by Asgari et al. [7, 8] and Kimothi et al. [9] utilized Word2Vec and Doc2Vec [23] respectively to demonstrate that useful low-dimensional and robust representations generated in an unsupervised fashion can support machine learning tasks over biological sequences. Other recent studies provide different methods to capture sequence [10–12] and structural information [13], generating vector encodings that were successfully utilized for structural similarity prediction and transmembrane prediction.

### 1.1 Contribution

This paper concerns learning of tailored word-based embeddings for molecular sequences to support sequence comparison without the need for sequence alignment. As with other embedding methods, our technique also requires as input a set of words or *k-mers* extracted from the sequence. The effect of learning is to capture the information implicit in these *k-mers*, markedly reducing the dimension from the full bag-of-words representation while ensuring that related *k-mer* groups have proximal representations in the embedding space. We improve these associations by the addition of class information through supervision, and subsequently through the use of supervision over defined class hierarchies. In this way, we may compute sequence similarity accurately over vectors within a lower dimensional subspace, while ensuring that the calculation relies on features pertinent to the problem at hand, here reflecting some biological grouping or functional relationship.

In the earlier bioinformatics applications ([7], [9]), *k-mers* are embedded in a manner that reinforces their surrounding context, implicitly capturing the ‘semantics’ of these co-occurring terms. Here we implicitly adopt a distributional hypothesis for sequence *k-mers*, the view that commonly occurring *k-mers* are likely drawn from functionally related contexts as part of some region of a gene or protein, or as a constituent of some regulatory region. The nature of these relationships depends crucially on the type of the sequence and on the resolution implied by the choice of *k-mer* size, *k*. The addition of metadata to the training process both reinforces and complements the relationships inherent in the sequence representation. Such labels are commonly used to indicate function (as in the *Gene Ontology* categories [14]) or associations and may be used directly (see, for example, the protein-protein interaction work of [15]) or indirectly to support inference. Class information of this nature may be available implicitly in features or representations exploited by other methods, for example, in the BLOSUM scores employed by BLAST [16] and MMseqs2 [5], which summarise the alignments of hundreds of proteins.

In this paper, we propose two supervised approaches that capture meta information along with the sequence content, utilising both sources to generate biological sequence embeddings. These approaches—SuperVec and SuperVecX—are inspired by the text embedding methods Word2Vec [6] and fastText [17]. For convenience we will refer to SuperVec and SuperVecX jointly as SuperVec(X). These methods make supervised learning of sequence representations based on class information explicit, extending the earlier context-based ([7, 9]) approaches to incorporate additional labels or metadata associated with the sequences—and with the resulting *k-mer* set. Our hypothesis is that by infusing meta information in the learning process, the approach will yield sequence representation better suited to the task at hand, with members of the same class (even if they are divergent sequences—those who might otherwise exhibit a lower degree of shared context)—embedded proximally within the vector space. In SuperVec, this is achieved by joining two embedding models in the one framework. Here, the first enforces class supervision, while the second incorporates contextual information present in the sequence, achieved as before by extracting a set of *k-mers* from each sequence. Class information constrains the intra-class vectors to fall closer together within the vector space, which in turn induces class information in the embedding of the constituent *k-mer* sets.

SuperVecX incorporates class-label information in the *k-mer* embeddings, using the *kmers* of a sequence to predict the corresponding class label. The architecture is a simple linear neural network classifier that has a linear hidden layer and softmax layer at the output. Once trained, the embedding for a new sequence is obtained by averaging the vectors of its constituent *k-mers*. Because of its simple architecture, the approach is fast and scales effectively to large sets of sequences.

Although both SuperVec and SuperVecX are supervised techniques, there are some underlying differences between them:

- SuperVec uses both contextual information as well as the class-label information to learn sequence embeddings, whereas SuperVecX relies only on the class-label information. As we will show later, each of these methods may outperform the other depending upon the task at hand.
- The architecture of SuperVec is flexible and we can easily accommodate unlabelled sequences for training—unlike SuperVecX where the class label information is essential for training.

We demonstrate the utility of our proposed approaches for two tasks: (a) retrieval of homologous sequences and (b) protein classification. To apply these approaches to the retrieval task, we consider the protein family tag (retrieved from the PFAM database [18]) as the class-label for a given sequence. For the protein classification tasks, the class-labels are given implicitly. For example, for subcellular location prediction, the site of the protein within the cell (mitochondria, pathway, nucleus, and cytosolic) is considered as the class label for the sequence.

The homologous sequence retrieval task pertains to retrieval of sequences with a common evolutionary history from a database. Here, we take membership of the same protein family as a marker of homology, relying on the family definition provided in PFAM [18] for protein sequences. The exponential increase in the database size over the last decade or more has posed fundamental challenges for alignment-based techniques for large scale sequence comparisons. The computational complexity of these techniques increases with the size (in alphabets) of the database and input sequence. Our proposed techniques, reduce the dimension of any length sequence into a small fixed-length vector and are hence able to provide an order of magnitude improvement over alignment-based method in execution time for the homologous sequence retrieval task; the complexity of comparing two sequences in the proposed approaches is linear i.e., O(m) where *m* is the dimension of a sequence embedding. For both proposed methods, the number of classes affects the efficacy of the training process, ultimately limiting accuracy. To overcome these issues, we consider a range of partitions other than the original classes and construct a series of embeddings using the SuperVec(X) algorithm to better cover the space. This method, which we call Hierarchical SuperVec(X) or H-SuperVec(X), supports embeddings across feature spaces based on the class labels and their exclusive and exhaustive subsets.

Further, these methods can also be used as a filter to rapidly select a relevant subset of sequences from the large database, allowing methods with high precision to give the desired output. We call this strategy the Hybrid approach. The experimental results show that the Hybrid approach—H-SuperVecX+BLAST—is significantly faster than BLAST and gives similar performance for early recall levels for the retrieval task. For protein classification, we selected three different problems, namely toxin prediction [19], sub-cellular localization [20], and enzyme prediction [21]. Toxin and enzyme prediction are binary classification tasks, in which a protein is predicted as toxin/non-toxin or enzyme/non-enzyme using the sequences as input. Sub-cellular localization is a four-class classification problem, where the task is to predict one of the four (cytosolic, mitochondria, nucleus, and signal pathway) sub-cellular locations for a given protein sequence. We use our methods to generate sequence embeddings, which are then used as input to the classifier.

Experimental results illustrate that the proposed supervised embedding approaches provide a substantial improvement in retrieval performance vis-a-vis the embedding methods reported in [7] and [9]; indeed for some cases the improvement offered by SuperVec and SuperVecX is double the precison value obtained using [7] and [9] for many recall-levels. These results demonstrate that our proposed hierarchical approaches—H-SuperVec(X)—further improve over vanilla SuperVec(X) providing a gain of 6 − 40% for many precision-recall values. For protein classification tasks in most of the cases, our methods give an improvement of 2 − 5% over unsupervised embedding techniques.

While the representations provided by our model are used in this paper for the homologous sequence retrieval and protein classification tasks, these representations can be directly utilized for other bioinformatics applications, such as the prediction of protein-protein interactions.

In summary, following are the main contributions of the paper:

- We present a supervised approach—SuperVec—to learning embeddings for biological sequences. SuperVec provides the flexibility to utilize meta-information (like class labels) along with the contextual information present in the sequences to generate their embeddings.
- We present SuperVecX—an alternative supervised embedding technique for generating biological sequence embeddings. The SuperVecX architecture is motivated from a recently popularized document classification technique called fastText.
- We present a hierarchical supervised approach that is designed specifically for the sequence retrieval task. We call this approach either H-SuperVec or H-SuperVecX depending upon the underlying embedding method—respectively SuperVec or SuperVecX.
- We show that our approaches provide a faster alternative to alignment-based methods like BLAST and MMseqs2 for the sequence retrieval task, providing an order of magnitude speedup in querying time.
- We show that our approaches can be used as a pre-processing filter prior to the application of a slower, high precision method for sequence retrieval tasks over a smaller dataset—allowing these results to be obtained far more rapidly.

In the next section, we give a more detailed introduction to the key approaches in Representation Learning, Word2Vec and Doc2Vec, providing the building blocks for the development of SuperVec and H-SuperVec in the subsequent sections.

## 2 Embedding models for text documents

In this section, we describe the text embedding approaches Word2Vec, Doc2Vec and fastText, that act as building blocks for the proposed supervised biological sequence embedding methods that follow in section 3.

The fundamental principle of Word2Vec [6] lies in the distributional hypothesis [22]: co-occurring words also share a semantic relationship. Word2Vec captures co-occurrence information in word embeddings by employing a simple classification task wherein a word is predicted based on its context (nearby words), and the representations are learned in a manner such that words with similar meanings appear proximal in the embedding space. Follow up works [23–25] extended the idea to include learning of embeddings for the whole document, based on suitable combinations of constituent word embeddings.

Asgari et. al. [7] introduced BioVec, an adaptation of the Word2Vec framework to learn embeddings for molecular sequences. Here the sequence embedding was obtained by the sum of the embeddings of all the *k-mers* present in the sequence. BioVec does not preserve the *k-mer* ordering of the sequence. Kimothi et.al. [9] addressed this limitation through Seq2Vec [9], which relies on the Doc2Vec [23] architecture to provide a direct embedding for the sequence.

While the application of Word2Vec and Doc2Vec to biological sequences is rather straightforward, the insights obtained are not. Both of these studies confirmed the utility of these embeddings in capturing relevant features of the protein sequences –- initially using BioVec and through subsequent improvements in Seq2Vec. The BioVec study also provided a valuable insight regarding the relationship among *k-mer* embeddings and the biochemical and biophysical properties of the sequence, the mass, volume, and charge. It was shown that *k-mers* with similar physicochemical properties form clusters in the embedding space. Further, in the Seq2Vec paper, we demonstrated that the use of the sequence tag during learning results in even better clustering of the sequence data—sequences belonging to the same family were more likely to be clustered together using Seq2Vec than with the BioVec model. Such representations have proven beneficial for the classification task.

### 2.1 Word2Vec architecture

**Fig 1:**
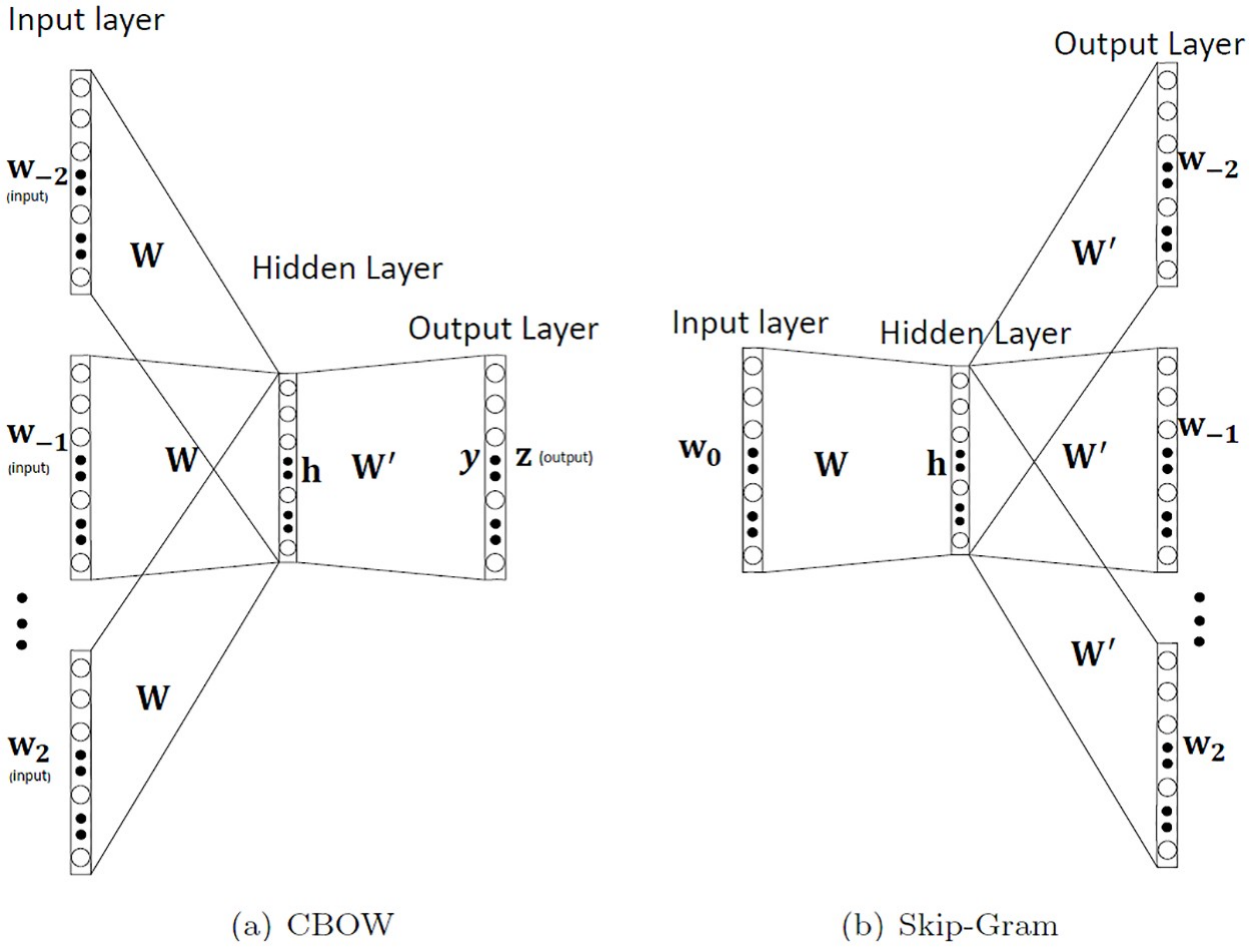
*Word2Vec* architecture: The figure shows two variants of word2vec architecture—CBOW and Skip gram [26] for a sample.

As mentioned before, the idea behind Word2Vec is that pairs of words that share a semantic relationship should be proximally located in the embedding space. Word2Vec achieves these embeddings through a fully-connected shallow neural network (NN) shown in Fig 1. Each node of this NN at input/output corresponds to a word in the vocabulary, and once the model is trained, the corresponding weight vector gives the embedding for the word in the vector space. Word2Vec comes in two different configurations reflecting the training method—namely the CBOW (Continuous Bag of Words) and the Skip-gram. In the CBOW architecture, the model is trained by giving the context at the input to predict the word, whereas in the Skip-gram architecture, the word is provided at the input to predict the context. Fig 1 shows these architectures for the sample *w*_−2_, *w*_−1_, *w*_0_, *w*_1_, *w*_2_. Here, *w*_0_ denotes the word at the center and the *w*_*i*_ (*i*≠0) denote the nearby context words. The subscript *i* denotes the position of words relative to the central word, *w*_0_. Inputs to the NN are here a one-hot-encoding of the word(s), as shown in small bold letters in the figure.

For the CBOW, the embeddings for words in the corpus are obtained by maximizing the joint probability of a word, *w*, conditioned on its context, *C*, for all samples in the corpus. The conditional probabilities for each sample are assumed to be independent of each other, and hence the joint probability for all samples can be written as ∏_*w*∈*Vocab*_ P*r*[*w* | *C*]. Maximizing ∏_*w*∈*Vocab*_ P*r*[*w* | *C*] is the same as minimizing its negative log-likelihood, so the overall loss function for Word2Vec is written as

$$\begin{array}{c} \begin{array}{c} J\left(W\right)=\sum_{w\in Vocab}-\log Pr[w\;|\;C]. \end{array} \end{array}$$ (1)

The above optimisation problem can be solved using the standard gradient descent approach. However, to increase the computational efficiency of the algorithm, Mikolov et al. in [27] use a negative sampling method that essentially modifies the original objective function so that each training sample only updates a few weight vectors.

### 2.2 Doc2Vec architecture

The Doc2Vec model essentially inherits the advantages offered by the Word2Vec model and generates a low dimensional representation of each document and its constituent words. These representations can then be used to retrieve documents from a collection. The main difference in the architecture of Doc2Vec and Word2Vec lies in the use of a document-tag along with the underlying words. The Doc2Vec architecture thus requires additional nodes to specify the document-tag as input. However, the output layer remains unchanged, and after training is completed, the Doc2Vec model yields both word embeddings and embeddings for the documents.

### 2.3 fastText architecture

Bojanowski et al. [28] extended the continuous skip-gram model to take account of sub-word information in learning vector representations for character *n*-grams. These representations are in turn added together in the construction of the word representation. For example, the word “*task*” is represented through the character n-grams “*tas, ask*” and the word itself. The representation of an entire document can be obtained by simply adding the representations for each of the words. The idea of using character *n* grams was used in yet another embedding method called fastText [17], which may be used to generate the document representation. Additionally, fastText also incorporated document labels to achieve the word embeddings. Like other embedding methods, fastText is also based on a shallow neural network and provides a rapid training performance. The proposed supervised embedding method SuperVecX is inspired by the fastText architecture.

Having introduced the background to our work and the components which underpin our model, we now explain in detail the architecture and operation of SuperVec, SuperVecX, and their hierarchical versions H-SuperVec and H-SuperVecX.

## 3 Proposed approaches: SuperVec and superVecX

In this section we will consider our supervised sequence embedding approaches SuperVec and SuperVecX. For convenience, we may refer to these methods jointly as SuperVec(X). As noted earlier, providing sequence labels while training may lead to a model that captures both a macro-level pattern from the class labels and the micro-level contextual information present in the protein sequences themselves.

We first discuss SuperVec and then SuperVecX, before introducing a new approach which utilizes the fact that SuperVec(X) may generate diverse embedding models when trained with a diverse set of classes. These sets may be obtained by partitioning classes randomly, hence generating a tree-like structure (Fig 4). The diversity of sequence embeddings generated from these models offers a range of query-subject distances, which may be processed jointly to give a better estimate of the similarity between the query-subject pair. We call these approaches Hierarchical SuperVec or H-SuperVec for SuperVec and H-SuperVecX for SuperVecX respectively. Jointly we will refer to these approaches as H-SuperVec(X).

The results presented later in this paper indicate that the use of label information during training, together with an increased diversity of representation models markedly improves retrieval performance while continuing to offer good computational performance.

**Notations**: We denote the corpus of *N* protein sequences by $S=\{s_{1},s_{2},\ldots ,s_{N}\}$ and the vocabulary with *M* unique *k-mers* as $K=\{k_{1},k_{2},\ldots ,k_{M}\}$. Each sequence $s_{i}=[k_{i_{1}},k_{i_{2}},\ldots ,k_{i_{n_{i}}}]$ is an ordered list of *k-mers*. To avoid notation clutter we use *s*_*i*_ to also denote its tag. Each sequence in S belongs to one of the *L* classes *l*_1_, *l*_2_, …, *l*_*L*_ and *s*_*i*_ ∈ *l*_*k*_ means *l*_*k*_ is the label for sequence *s*_*i*_. Finally, the embeddings of the *k-mer* *k*_*i*_ and the sequence *s*_*i*_ are denoted **k**_*i*_ and **s**_*i*_ respectively.

### 3.1 SuperVec

**Fig 2:**
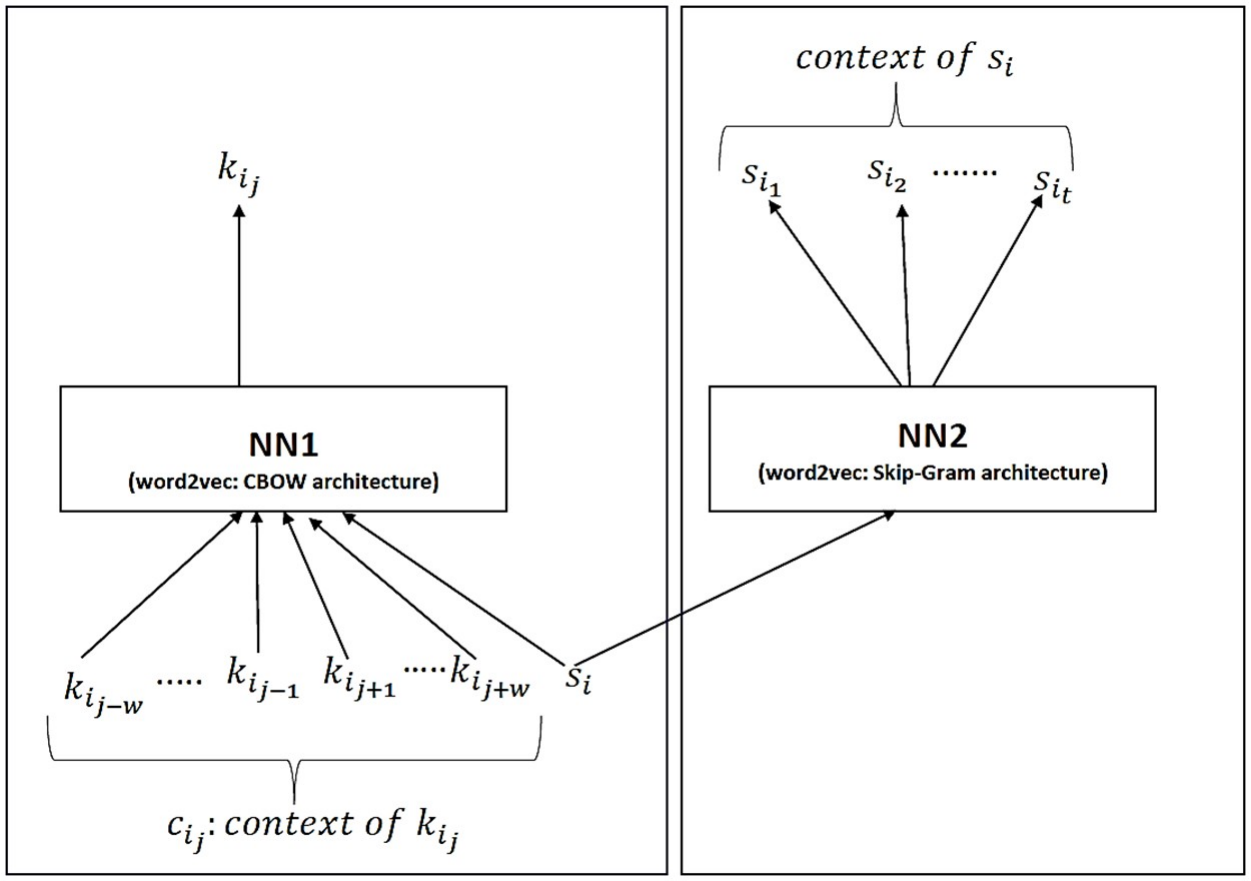
SuperVec model: NN1 and NN2 architectures are shallow neural networks with respectively the CBOW and the Skip-gram variant of the Word2Vec model. The context of $k_{i_{j}}$ comprises its nearby words and is denoted $k_{i_{j-w}},\ldots .,k_{i_{j-1}}$, $k_{i_{j+1}}....k_{i_{j+w}}$. The context of the *s*_*i*_ are sequences which have the same label as *s*_*i*_.

The SuperVec architecture consists of two Word2Vec units combined so that it can jointly incorporate the class label and the contextual information contained in the sequences. As shown in Fig 2, NN1 is a CBOW configuration of Word2Vec that generates an embedding for sequences using the contextual information, while NN2 follows a skip-gram configuration of Word2Vec to help incorporate the class level information. NN1 requires a word-context pair for training, while NN2 uses a sequence and a set of similarly labeled sequences. Note that NN1 forces the embeddings of the words that co-occur together along with the sequence embedding, while NN2 further constrains sequences having the same labels to be close to each other. Achieving embeddings informed by context *and* class information requires the use of *both* networks. Here we couple NN1 and NN2 by sharing the sequence embedding between them.

#### 3.1.1 Optimization problem formulation

To train the SuperVec model, i.e., to learn its parameters, two prediction tasks are performed, one by each of its sub-networks. Both networks essentially perform word(context)/context(word) prediction, although the meaning of context and word differs between them, as seen in Table 1 below; here we see the sample *kmer* $k_{i_{j}}$, its context $C_{i_{j}}$, and the corresponding sequence tag *s*_*i*_, and the relationships between them.

**Table 1:** context-word pairs for NN1 and NN2.

Network	Architecture	context	word
NN1	CBOW	*s*_*i*_ and $C_{i_{j}}$	$k_{i_{j}}$
NN2	Skip-Gram	$s_{i_{1}},s_{i_{2}}...s_{i_{t}}\in l_{k}$; given *s*_*i*_ ∈ *l*_*k*_	*s*_*i*_

Since the sub-networks share the sequence embedding task, the parameters of these networks influence each other. Mathematically, the coupling of these sub-networks means solving a joint optimization problem with the overall loss function, a linear combination of those for NN1 and NN2.

$$\text{J}(S,K)=\sum_{s_{i}\in S}\sum_{j=1}^{n_{i}}\left(\underset{NN1}{\underset{︸}{-\text{log}\;\text{P}r\left[k_{i_{j}}|C_{i_{j}},s_{i}\right]}}-\gamma \sum_{z\in I_{i}^{+}}\underset{NN2}{\underset{︸}{\text{log}\;\text{P}r\left[s_{z}|s_{i}\right]}}\right).$$ (2)

Here *γ* controls the balance between class information and contextual information. The matrices $K={\left[k_{1},\;k_{2},\ldots ,k_{M}\right]}^{T}\in R^{M\times n}$ and $S={\left[s_{1},s_{2},\;\ldots ,s_{N}\right]}^{T}\in R^{N\times n}$ denote the embeddings for *k-mers* and sequences respectively. We define $I_{i}^{+}$ to be the set of indices of sequences which have the same label as *s*_*i*_. The conditional probability for NN1 in Eq (2) can be computed by a softmax function, but considering the large number of *k-mers* and the computational burden involved, we approximate it using hierarchical softmax (HS) [29]. To compute the second part of Eq (2), we use *negative sampling* [27] where for any given sequence we try to maximize the probability of some selected positive samples as opposed to others. For example, in a context/word pair, all the words in the context can be treated as positive samples and the remaining words in the vocabulary as negative samples. In our case, for any input sequence *s*_*i*_, sequences whose index lies in $I_{i}^{+}$ are seen as positive samples, whereas the remaining sequences are seen as negative samples. We denote the set of indices of the negative samples as $I_{i}^{-}$. The second part of Eq (2) can then be approximated as

$$\begin{array}{c} \log\sigma (\langle s_{z},s_{i}\rangle )+\sum_{r\in I_{i}^{-}}^{}\log\sigma (-\langle s_{r},s_{i}\rangle ), \end{array}$$ (3)

where $\sigma (x)=\frac{1}{1+\exp(-x)}$ is the sigmoid function. Maximizing the first part of the Eq (3) maximizes 〈**s**_*z*_, **s**_*i*_〉 and therefore reduces their cosine distance in the embedding space. Similarly, maximizing *σ*(−〈**s**_*r*_, **s**_*i*_〉) translates into maximizing the cosine distance between **s**_*i*_ and **s**_*r*_. As defined above, the *s*_*z*_ have the same label as *s*_*i*_ whereas the *s*_*r*_ have a different label. Maximizing Eq (3) therefore forces the sequences from the same class to be mapped closer in the embedding space, while other sequence pairs are pushed apart. Employing negative sampling thus yields embeddings with low intra-class and high inter-class separation, a mapping well suited to the retrieval task. Replacing the second part of Eq (2) with its *negative sampling* expansion, the final loss function for SuperVec is given as

$$\begin{array}{cc} J^{}\left(S,K\right)= & \sum_{s_{i}\in S}\sum_{j=1}^{n_{i}}[-\log Pr[k_{i_{j}}\;|\;C_{i_{j}},\;s_{i}]\\ & -\gamma \sum_{z\in Z_{i}^{+}}(\log\sigma (\langle s_{z},\;s_{i}\rangle )+\sum_{r\in Z_{i}^{-}}\log\sigma (-\langle s_{r},\;s_{i}\rangle ))]. \end{array}$$ (4)

Computing the second part of Eq (4) requires a very large number of operations—the *number of positive samples* × *number of negative samples*—for each case. To mitigate this computational burden, we use only a few randomly selected positive and negative samples for any given sequence. In Eq (4), $Z_{i}^{+}$ and $Z_{i}^{-}$ are sets consisting of the indices randomly chosen from the positive indices, $I_{i}^{+}$, and from the negative indices, $I_{i}^{-}$, respectively. Note that we sample positive as well as negative samples to further speed up the training process.

#### 3.2.1 Parameter learning

The parameters of SuperVec govern the construction of the embeddings of the *k-mers* and the sequences. The process of learning the parameters involves a training process similar to that used for the Word2Vec framework. The parameters are initialized randomly and then modified for each sample to reduce the value of the loss function. The sample here consists of the *k-mer*, its context, and the corresponding sequence tag. As explained before, SuperVec employs two prediction tasks for each selected sample. In these prediction tasks, for any context/word at the input, the parameters are modified to maximize the probability of word/context at the output by updating the values of parameters using gradient descent. After sufficient iterations, we obtain a trained network which retains the relevant information—in this case, the contextual and class information. The update equations and the derivation of the gradient for **s**_*i*_, $k_{i_{j}}$ and other parameters of SuperVec over J(**S**, **K**) are provided in the S1 Appendix. Once the model is trained, it can be employed in learning the representation of any new sequence. Note that new sequences do not require any label information: as discussed below, we use only NN1 for learning this representation.

#### 3.1.3 Inference

Computing the representation of a new sequence by passing it through the trained model constitutes the inference step. Unlike the training of SuperVec, since its inference step does not utilize the sequence labels, only one of the sub-networks of SuperVec, i.e., NN1 (refer to Fig 2), is used. Although the standalone NN1 only uses contextual information, we expect that since NN1 and NN2 are coupled and jointly trained, NN1 will also capture the class information to some extent and that this will eventually influence the representation learned in the inference step. While the representation for a sequence is computed during the inference step, all the parameters of NN1 remain unchanged except for the sequence vector, which is initialized with zeros. This vector is updated iteratively following a gradient descent approach similar to the training stage.

### 3.2 SuperVecX

SuperVecX is inspired by a recent document classification method called fastText [17] that can also be used to learn sentence embedding. Unlike unsupervised word vectors, fastText provides word embeddings that can be averaged to obtain a fruitful sentence representation. Since fastText is a linear model, training is substantially faster than the other deep learning approaches used for sentence classification. The computational efficiency offered by fastText is particularly attractive for applications in bioinformatics, which may involve millions of sequences in the training set. Below we briefly discuss the SuperVecX architecture, its parameter learning, and the inference procedure.

#### 3.2.1 Model description and parameter learning

**Fig 3:**
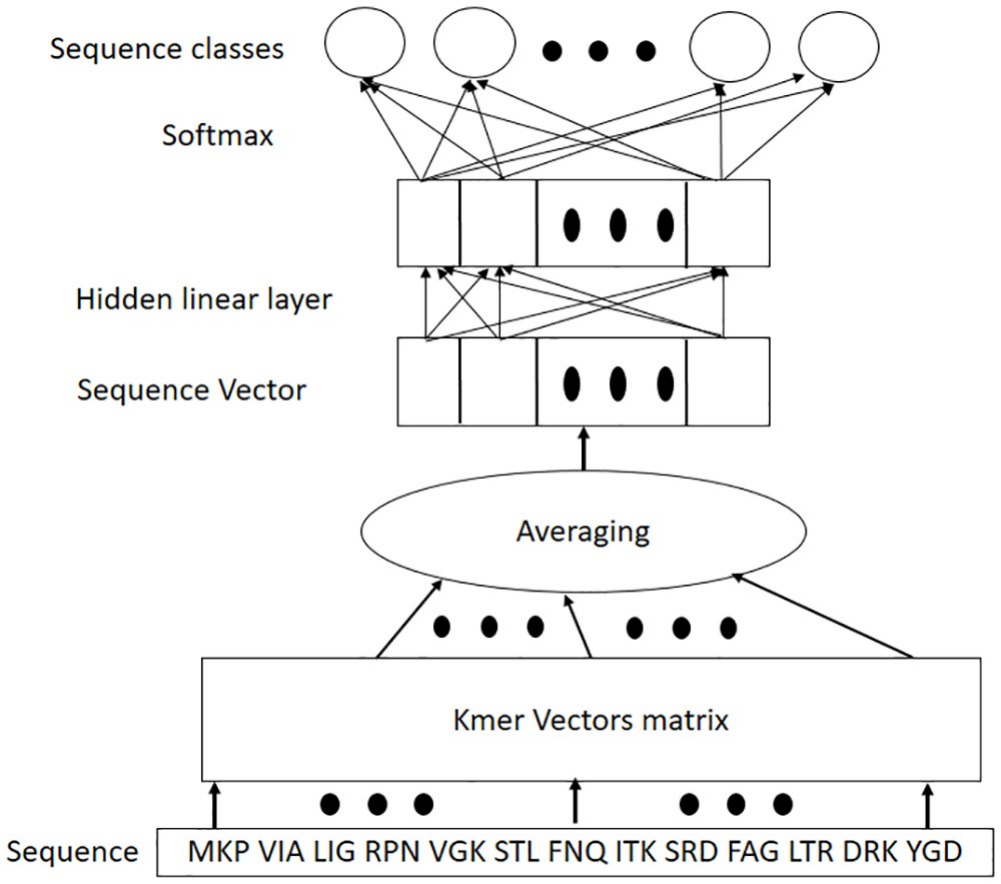
SuperVecX: A supervised method for generating sequence embeddings [30].

The architecture of SuperVecX is shown in Fig 3. It is a shallow neural network consisting of an input, a linear hidden layer, and an output layer. The model is trained by presenting a sequence from the corpus to the network to predict its class. Note that the sequence is given as a list of its sub-sequences (*k-mers*). The input to the model is obtained by averaging the vectors corresponding to the *k-mers* in the input list. These vectors constitute a *k-mer* matrix that is randomly initialized at the start of training. At the output layer, the softmax function is applied to get the probability distribution of all the classes for the given input sequence. The overall objective of the training process is to maximize the probability of the classes given their corresponding sequences, hence learning the model parameters (the *kmer* vectors and the weight matrix).

#### 3.2.2 Inference

The inference step of SuperVecX is simple as it requires only the embeddings of the *k-mers*. Once the model is trained, the embedding for a new sequence is obtained by averaging the embeddings of its constituent *k-mers*. The efficacy of the inference step is evaluated with respect to the task for which the learned representations are employed. In this paper, we consider retrieval as one of the downstream applications. Here it is expected that the information learned by the trained model will be exploited in the representation of the new sequence.

The comparison of unsupervised methods and SuperVec(X) results for retrieval tasks confirms our hypothesis that incorporating label information in the training process helps in generating useful sequence embeddings for retrieval purposes. Although SuperVec and SuperVecX perform better than other representation learning methods (Seq2Vec [9], BioVec [7]) for a number of retrieval tasks, we observe that their retrieval performance deteriorates with an increasing number of classes.

For SuperVec, with the increase in the number of classes, the constraints enforcing interclass and intraclass separation necessarily increase in number. Satisfying this set of constraints may prove difficult, reducing the efficacy of the training process and ultimately leading to a deterioration in retrieval performance. Although the interclass and intraclass constraints are not imposed explicitly for the SuperVecX, these constraints are enforced implicitly following the classification task involved in its training. The other important observation to note here is that the interclass and intraclass distances of a set of sequences change when we generate sequence embeddings using SuperVec(X) models trained on a diverse set of classes. This observation implies that we get a diverse embedding of a sequence when generated through multiple models.

**Fig 4:**
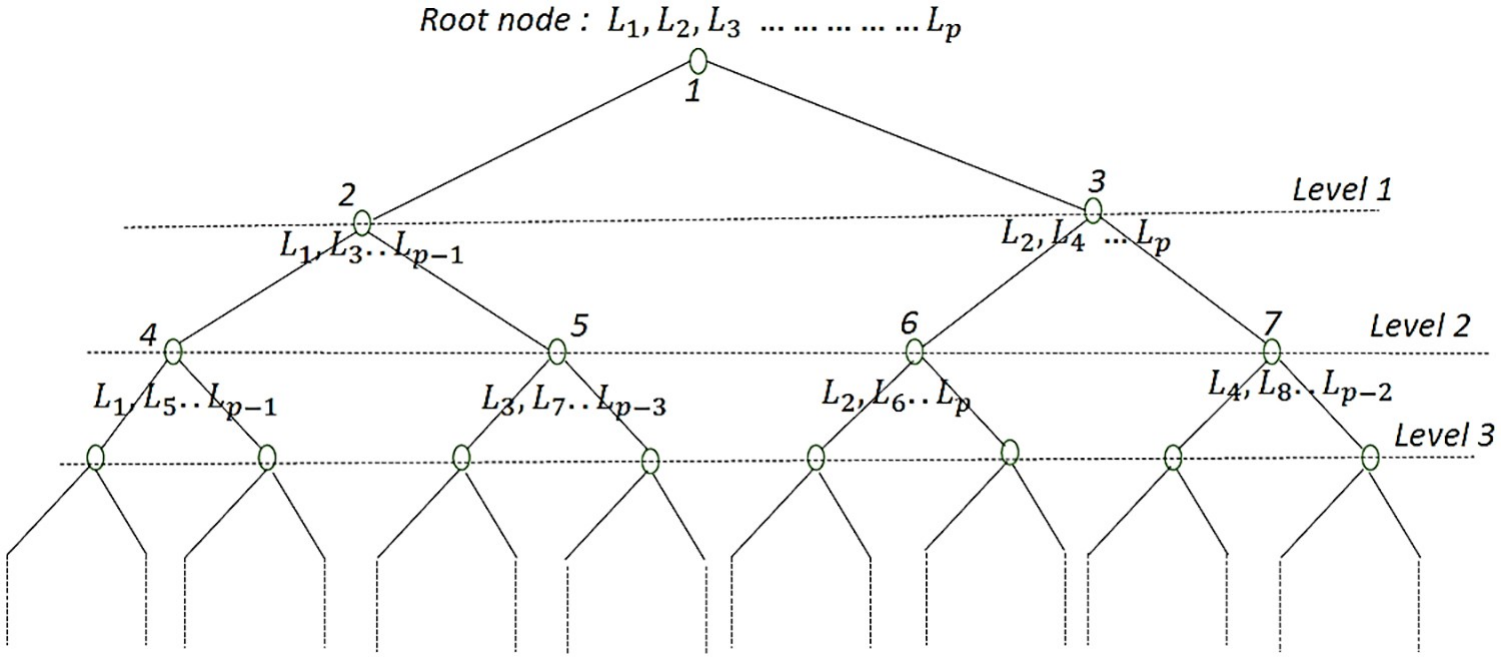
An example binary tree for H-SuperVec(X): A hierarchical structure obtained by partitioning the class labels of each parent node into equal size subsets. The root node is assigned *p* class labels (*L*_1_, *L*_2_….*L*_*p*_) and their corresponding sequences. In this example we assume that *p* is an even number; the right child of the parent node is assigned the even and the left child is assigned the odd indexed labels selected from those assigned to the parent node.

Keeping these observations in mind, we propose a hierarchical approach that improves the retrieval results by computing a better estimate of the query-subject similarity. We call this proposed method H-SuperVec or H-SuperVecX for SuperVec and SuperVecX respectively, or collectively as H-SuperVec(X). In this approach, we work with a binary-tree obtained by partitioning a set of classes at each parent node (refer Fig 4). Once the tree is created, the SuperVec(X) model is trained for each of these nodes. Following this approach gives us multiple trained models, which can be used to generate many observations of the same quantity (here the query-subject distance). These observations are processed jointly to get a better estimate of query-subject similarity.

We describe the H-SuperVec(X) model in detail in following section.

## 4 Hierarchical SuperVec(X)

H-SuperVec(X) exploits the fact that we can generate multiple, diverse embeddings for a given sequence using multiple SuperVec(X) models trained on sequence data belonging to a diverse set of classes. Note that for each query-subject (database sequence) pair, each SuperVec(X) model results in a distance computation corresponding to that model. This distance is used as a proxy to measure the similarity between two sequences. Each SuperVec(X) model introduces some noise in the embeddings it generates and hence in the distance computed for any query-subject pair. Processing the query-subject distances obtained from different SuperVec(X) models together reduces the overall noise and gives us a better estimate of query-subject similarity. We utilize this fact in H-SuperVec(X) and apply it to the same retrieval task. Applying H-SuperVec(X) for retrieval tasks involves the following steps:

- Form a Hierarchical Structure: First, we assign all of the class labels and their corresponding sequences to the root node. The root node is then split into two child nodes by randomly partitioning its associated class labels into equal halves. These child nodes are further partitioned, following the same process for each node until we are left with leaf nodes, each associated with a single class label. An example of such a hierarchical tree is provided in Fig 4.
- Train a SuperVec(X) model for each node of the above tree: these models can subsequently be used to generate embeddings for a new sequence.
- Assign weights to each node: As we traverse down the tree, SuperVec(X) is successively trained with fewer classes, leading to an increase in noise in the computed query-subject distance. To get a better estimate of query-subject similarity, we apply a simple linear model (weighted sum) over the distances computed at each node. For query (*q*), the similarity is estimated as the weighted sum:
  $$\begin{array}{c} d=\sum_{i\in nodes}w_{i}d_{i}, \end{array}$$ (5)
  where *d*_*i*_ is the query-subject distance computed at the *i*^*th*^ node and *w*_*i*_ is the weight assigned to node *i*. Since noise increases as we traverse down the tree, the largest weight is assigned to the root node, with node weights decreasing as we traverse toward the leaves. The weight magnitudes are constrained by three conditions: (i) the weights are positive, *w*_*i*_ > 0, ∀*i*; (ii) the weights at child nodes are assigned based on the probability (computed at parent node) with which a query can be assigned to the child nodes. For our experiments we use nearest neighborhood classification for assigning probabilities; (iii) the weights sum to one, i.e., ∑_*i* ∈ *nodes*_ *w*_*i*_ = 1.
- Retrieve sequences: Once we build a hierarchical tree and train the SuperVec(X) model for each node, the retrieval task is performed as follows. First, we learn embeddings for database sequences using the SuperVec(X) model at each node. For a new query sequence, multiple embeddings are generated corresponding to the nodes of the tree using the inference step. Pairwise query-subject distances are then calculated for each node using Eq (6). These distances are finally combined as a linear sum to give an estimate of similarity between the query-subject pair.
  The results are returned in descending order of their similarity with the query sequence.

**Fig 5:**
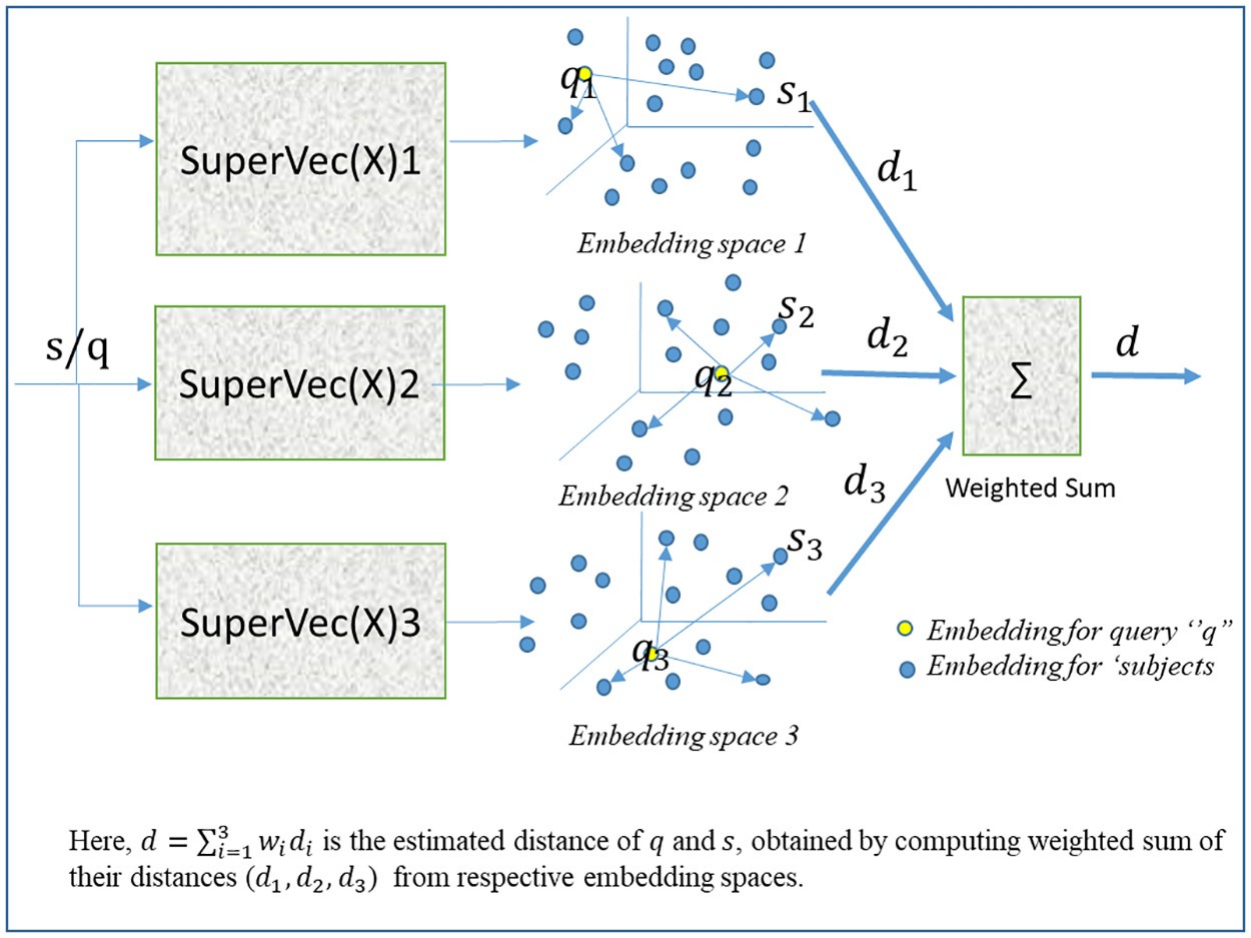
SuperVec(X) 1/2/3 are the SuperVec(X) model trained for node 1, 2 and 3 respectively. **q**_*i*_ and **s**_*i*_ are the embedding of a query (*q*) and subject (*s*) and *d*_*i*_ is the distance of query-subject pair computed at *i*^*th*^ node, *d* is the similarity score for *q* and *s*.

Since the weight assigned to each node is reduced as we traverse down the tree, the contribution of the nodes in the computation of similarity between query-subject pair also decreases. We empirically determined that working with a one or two-level tree produces consistently better retrieval results. For our experiments, we chose a tree with only one level, i.e., the tree having root and its child nodes. We demonstrate the mechanism followed by the H-SuperVec(X) method to estimate the distance of query-subject pair in Fig 5, where, SuperVec(X)1, SuperVec(X)2 and SuperVec(X)3 are the models for root and first-level nodes respectively. Each of these models is utilized to obtain the query-subject distance; the distance computed at *i*^*th*^ node and hence through *i*^*th*^ model is given as,

$$\begin{array}{c} d_{i}=1-\frac{\left\langle q_{i},s_{i}\right\rangle }{∥q_{i}∥∥s_{i}∥}. \end{array}$$ (6)

where **q**_*i*_ and **s**_*i*_ are the embeddings of query ‘q’ and a database sequence ‘s’ computed at *i*^*th*^ node.

For the experiments, we fix the node weight to be 0.5, the weights for other nodes are chosen following the process as discussed before. Employing hierarchical approaches H-SuperVec(X) for the retrieval task gives an improvement over other representation learning methods considered in this paper, including the naive SuperVec(X) methods; the results are shown in section 5.4.1.

## 5 Experiments and evaluation

We now demonstrate the quality of the embeddings generated through the proposed methods on (i) homologous sequence retrieval and, (ii) protein classification tasks. In the homologous sequence retrieval problem, the goal is to return a set of sequences from a database that are homologous to a given query. For the embedding based methods, we address the retrieval problem by employing the nearest neighbour search, where for a given query, the sequences from a database are retrieved based on the cosine-distance of query and database sequence embeddings. The protein classification tasks are concerned with predicting the class for a given protein sequence. Here, we apply the proposed embeddings on the toxin prediction, subcellular localization, and enzyme prediction tasks.

Below, we discuss the data, experimental design, and results for the sequence retrieval and protein classification tasks in turn.

### 5.1 Homologous sequence retrieval task

#### 5.1.1 Data

We use the subset of protein sequences from Swiss-Prot [7] and Uniref50 [31] databases for the evaluation of our approaches on the sequence retrieval task. For convenience, we call these datasets as Dataset1 and Dataset2, respectively. The total number of sequences in Dataset1 and Dataset2 is 150k and 1.1M, respectively, as indicated as the subscript in Swiss-Prot_150k, and Uniref50_1.1M.

The description of these datasets is as follows:

**Dataset1: Swiss-Prot_150k**
  The Swiss-Prot dataset consists of 324018 protein sequences, each uniquely annotated with one of the 7027 family labels. In this dataset, some families have the same functional description as in the PFAM database. For example, Chitin_synth_1 (PF01644) and Chitin_synth_2 (PF03142) are two different entries in PFAM, but both represent the chitin synthase enzyme. We merged such families into a single representative family leading to a reduction in the number of families to 6967. The families/classes present in this dataset differ considerably in their size; the largest family contains 3024 sequences, whereas many families are based on a single sequence.
  To create a reasonably sized training and test splits for experiments we chose the largest 200 families from this dataset, ensuring a minimum family size of at least 400 members, making our total dataset size to be of 150, 324 sequences.
**Dataset2: Uniref50_1.1M**
  Uniref50 database contains clusters with sequences that share 50% or more similarity; each cluster is represented by a consensus sequence also called a representative sequence. After pre-processing the data, we keep only those sequences that are annotated with only one “PFAM” entry. For experiments, we keep medium sized classes that contain 400 − 1000 sequences. There are 1866 such classes with a total of 11, 921, 50 sequences that comprises dataset2.
  A detailed description of both the datasets is given in the S4 Appendix.

#### 5.1.2 Experimental design

The experimental setup used to conduct the retrieval experiments in this paper is discussed below.

- **Setup 1:** This setup is designed to demonstrate the advantages of the supervised methods SuperVec, SuperVecX and their extension H-SuperVec and H-SuperVecX over unsupervised embeddings on the sequence retrieval task. Here, the database is constructed by choosing at random 60% of the sequences from each class (each family corresponds to one class). The remaining 40% of these sequences form a query set that is used to evaluate retrieval performance on the database. Retrieval based on a given query proceeds as follows. First, for each database sequence, an *n*-dimensional embedding is generated, yielding a database embedding space. For a new query, we first generate its embedding; then, we employ nearest-neighbor search in the database embedding space, returning sequences in descending order of cosine similarity using the nearest neighborhood search [32].
  Note that the process of generating embeddings for the sequences differs for each of the considered Representation learning (RepL) methods. For BioVec, sequence embeddings are generated by adding the corresponding *k-mer* embeddings; we use the *k-mer* embeddings provided by [7]. To generate the sequence embedding using the Seq2Vec or SuperVec approach, we first train them using the database sequences; note that unlike Seq2Vec, SuperVec also uses the database sequence label for training. Once these models are trained, the sequence vectors are generated by the inference step. For SuperVecX, the sequence embedding is obtained by adding the corresponding *k-mer* embeddings generated through the trained SuperVecX.

In this study, all experiments are performed using a commodity Linux workstation equipped with an Intel Core i7-4790K, 3.6GHz 8 core, 16 thread processor. The values of hyper-parameters–the *k*-mer size and the representation length of sequences for SuperVec and Seq2Vec–are kept same as given in [7], whereas the context size and supervision parameter *γ* are chosen based on the best retrieval results obtained on a database of largest 50 classes under setup 1. We also evaluated our methods for a few different values of *k* (3,4,5) and found that retrieval performance remains almost the same. The values selected for the SuperVec, SuperVecX and Seq2Vec hyper-parameters are shown in Table 2.

**Table 2:** Hyper-parameters for SuperVec, Seq2Vec and SuperVecX.

Parameter	Value
*k-mer* size	3
context size	1
Sequence representation length	100
*γ*	0.5

#### 5.1.3 Evaluation

The performance of the supervised embedding methods and their hierarchical versions on the homologous sequence retrieval task is evaluated through comparison with the other RepL approaches—BioVec and Seq2Vec—and with a standard alignment-based approaches, BLAST, and MMseqs2.

The evaluation metrics for the retrieval task are chosen as follows:

1. **Interpolated precision-recall values:** The interpolated precision value *p*_*interp*_(*r*) [33] at a recall level *r*, is defined as highest precision value found for any recall level *r*′ >*r*:
  $$\begin{array}{c} p_{interp}\left(r\right)=\underset{r^{{}^{\prime }}\geq r}{\text{max}}\;p\left(r^{{}^{\prime }}\right) \end{array}$$
  Here, *p*(*r*′) is the precision value at recall *r*′; *p*(*r*′) is defined as the ratio of the number of relevant subjects (database sequences) at recall *Rel*@*r*′ and the total number of subjects to be retrieved from the sorted list of subjects so as to output *Rel*@*r*′(*ER*_*Rel*@*r*′_). For example, for a query Q with label L, *p*(*r*′) is calculated as,
  $$\begin{array}{c} p\left(r^{{}^{\prime }}\right)=\frac{Rel@r^{{}^{\prime }}}{ER_{Rel@r^{{}^{\prime }}}}. \end{array}$$ (7)
  Here *Rel*@*r*′ is *r*′ of the database sequences (subjects) with label L. Note that the subjects are sorted based on their similarity to the query sequences, usually computed based on the alignment score. For RepL approaches, we use the cosine similarity of query and the subject to compute the similarity. The *Rel*@*r*′ is fixed for any search algorithm, a better search algorithm has a smaller *ER*_*Rel*@*r*′_, and hence gives higher *p*(*r*′).
2. **Querying Time:**
  The querying time is defined as the time required to retrieve the significant matches from the database for a given query. As discussed before, the retrieval process for RepL methods requires that we first train the model, which is then used for generating the representations for the database and subsequent query sequences followed by the nearest-neighbor search. This training process is computationally intensive but is required only once for each model. The training time for SuperVec for ∼90*k* training sequences is ∼28 hours, and there remains scope to improve this performance by using a distributed training framework for our approach. The idea is to use multiple GPUs on a cluster to train in a distributed fashion and then use all reduce operation to compute the full gradient. We expect the training time to linearly decrease as we increase GPU’s. Since SuperVecX is a linear classification model, it is sufficiently fast to work with much larger datasets; it takes around ten minutes for training over ∼90*k* sequences. We compute the querying time as the sum of the time taken to generate the sequence embedding and the time required to produce the list of nearest neighbor(s) for a given query. For BLAST and MMseqs2, we report the querying time as the time required to return the list of database sequences for a given query.

#### 5.4.1 Results and discussion

In this section, we present the results obtained on the retrieval task for SuperVec, SuperVecX, their corresponding hierarchical version—H-SuperVec and H-SuperVecX and other considered methods. For convenience, to refer both of these methods together we write SuperVec(X) and H-SuperVec(X) respectively. We first show that SuperVecX outperform unsupervised embeddings on sequence retrieval tasks, then we present results for hierarchical approaches —H-SuperVec(X) showing their superiority over SuperVec(X). Finally, we show that these approaches can be used to pre-filter the database that is further processed through a high accuracy approach like BLAST thus providing the best of both alignment and alignment-free approaches, i.e., accuracy and speed.

**Supervised vs unsupervised embeddings**. To demonstrate the effect of supervision on retrieval performance, we initially consider a retrieval task over a small database (two-classes) from dataset1. The averaged result over 100 randomly chosen pairs is provided in Table 3. The result shows that the proposed supervised embedding approaches outperform the unsupervised approaches over this task. As the RepL approaches fall broadly under the umbrella of alignment-free methods, we also consider the performance of BLAST a most widely used of the alignment-based approaches on these tasks. The results show that SuperVec and SuperVecX outperforms BLAST for the two-class database problems.

**Table 3:** Retrieval results for 100 random pairs: Average interpolated precision values at ten recall levels computed for 100 random pair of classes. All of these pairs differ in the number of database and query sequences. The precision value shown at particular recall level below is averaged over the chosen 100 pairs.

Methods	**Recall Levels**				
	0.1	0.2	0.3	0.4	0.5
BioVec	0.9079 ± 0.087	0.8817 ± 0.101	0.8573 ± 0.112	0.8329 ± 0.124	0.8066 ± 0.134
Seq2Vec	0.9494 ± 0.034	0.9258 ± 0.05	0.904 ± 0.064	0.8828 ± 0.076	0.8616 ± 0.085
SuperVec	0.9899 ± 0.009	0.9882 ± 0.011	0.9868 ± 0.013	0.9853 ± 0.014	0.9836 ± 0.016
SuperVecX	0.9339 ± 0.08	0.9261 ± 0.1	0.9216 ± 0.10	0.9176 ± 0.11	0.9146 ± 0.11
BLAST	0.9705 ± 0.046	0.9562 ± 0.082	0.9416 ± 0.105	0.9322 ± 0.115	0.9208 ± 0.133
Methods	**Recall Levels**				
	0.6	0.7	0.8	0.9	1
BioVec	0.7764 ± 0.142	0.7458 ± 0.147	0.7109 ± 0.149	0.6617 ± 0.143	0.5520 ± 0.082
Seq2Vec	0.8380 ± 0.094	0.8110 ± 0.102	0.7778 ± 0.108	0.7320 ± 0.111	0.6247 ± 0.095
SuperVec	0.9815 ± 0.018	0.9788 ± 0.021	0.9749 ± 0.025	0.9675 ± 0.032	0.9279 ± 0.06
SuperVecX	0.9095 ± 0.126	0.9038 ± 0.13	0.8941 ± 0.14	0.8782 ± 0.15	0.8440 ± 0.17
BLAST	0.9084 ± 0.153	0.8918 ± 0.180	0.8547 ± 0.214	0.7896 ± 0.259	0.0485 ± 0.136

**Fig 6:**
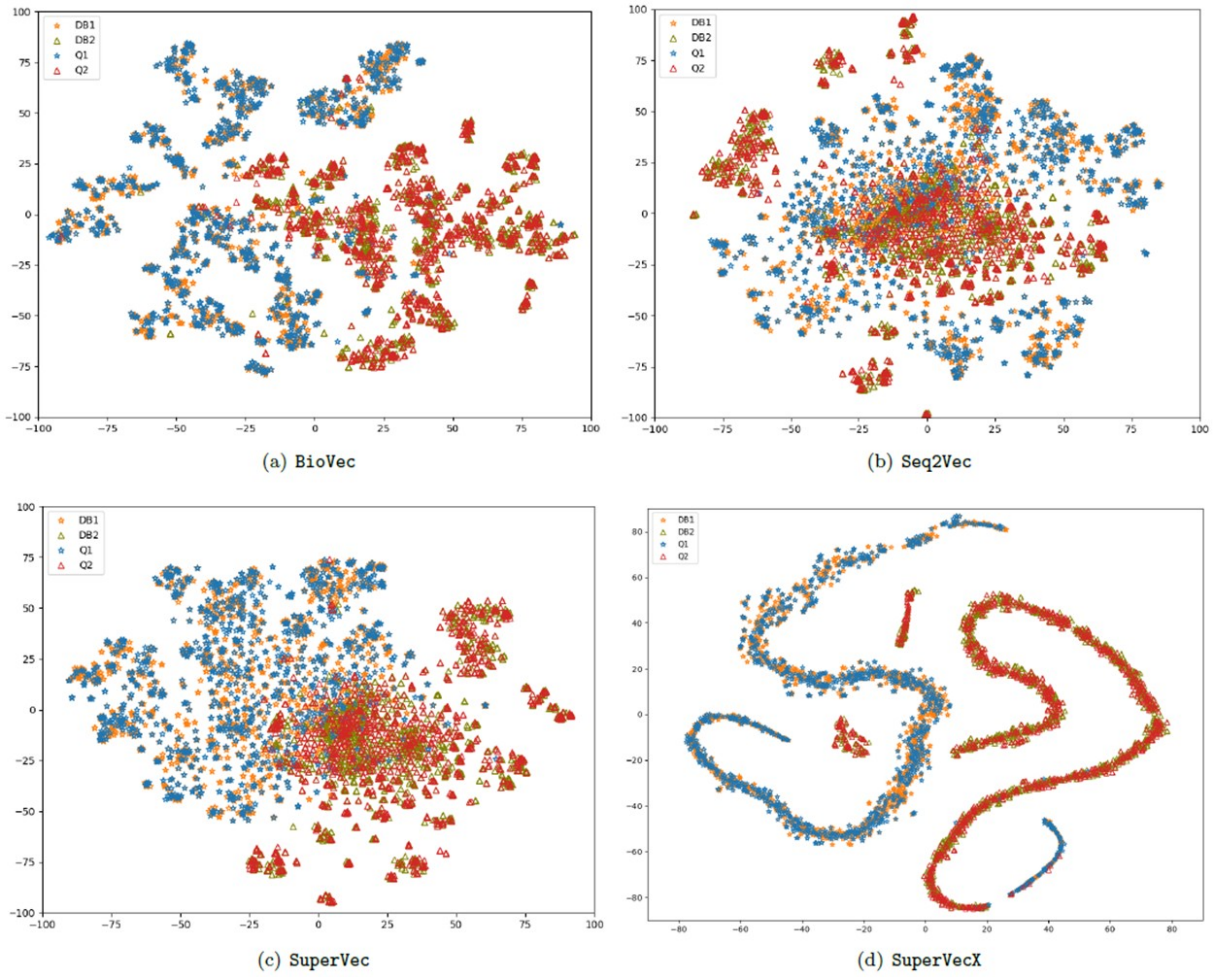
t-SNE plots: The mapping of database and query embeddings generated through BioVec, Seq2Vec, SuperVec and SuperVecX approaches. DB1, DB2 denotes the database sequences and Q1, Q2 denotes the query sequences from class1 and class2 of dataset1.

We also visualize and compare the embeddings of the database and query sequences generated through our proposed supervised approaches and existing unsupervised methods in two-dimensional space. We use t-SNE [34] tool to provide the two-dimensional mapping of the embeddings. The plots provided in Fig 6 are obtained for the largest two classes of dataset1; the enlarged figures for these plots are provided in S1 Fig.

From the plots in Fig 6, we observe that:

- BioVec generated database embeddings are well-separated by class, but form small groups within each class. Seq2Vec provides (relatively) better intraclass organisation than BioVec, but the two classes are merged to a great extent. SuperVec database embeddings show a better intraclass and interclass separation than the other methods, albeit with some overlap at the boundary.
- For a new query, the presence of the relevant subjects (here the database sequences from the same class) decreases as we increase the neighborhood size. Thus, we expect to see a decrease in the precision values for increasing recall levels. Analyzing the plots, we can infer that such an effect will have a stronger impact on the retrieval performance of Seq2Vec and BioVec when compared to SuperVec, especially for late recall levels. For both database and query sequences SuperVecX provides well-separated embeddings in the vector space, which will thus be expected to give the best result among all considered RepL methods for the chosen classes.

**Fig 7:**
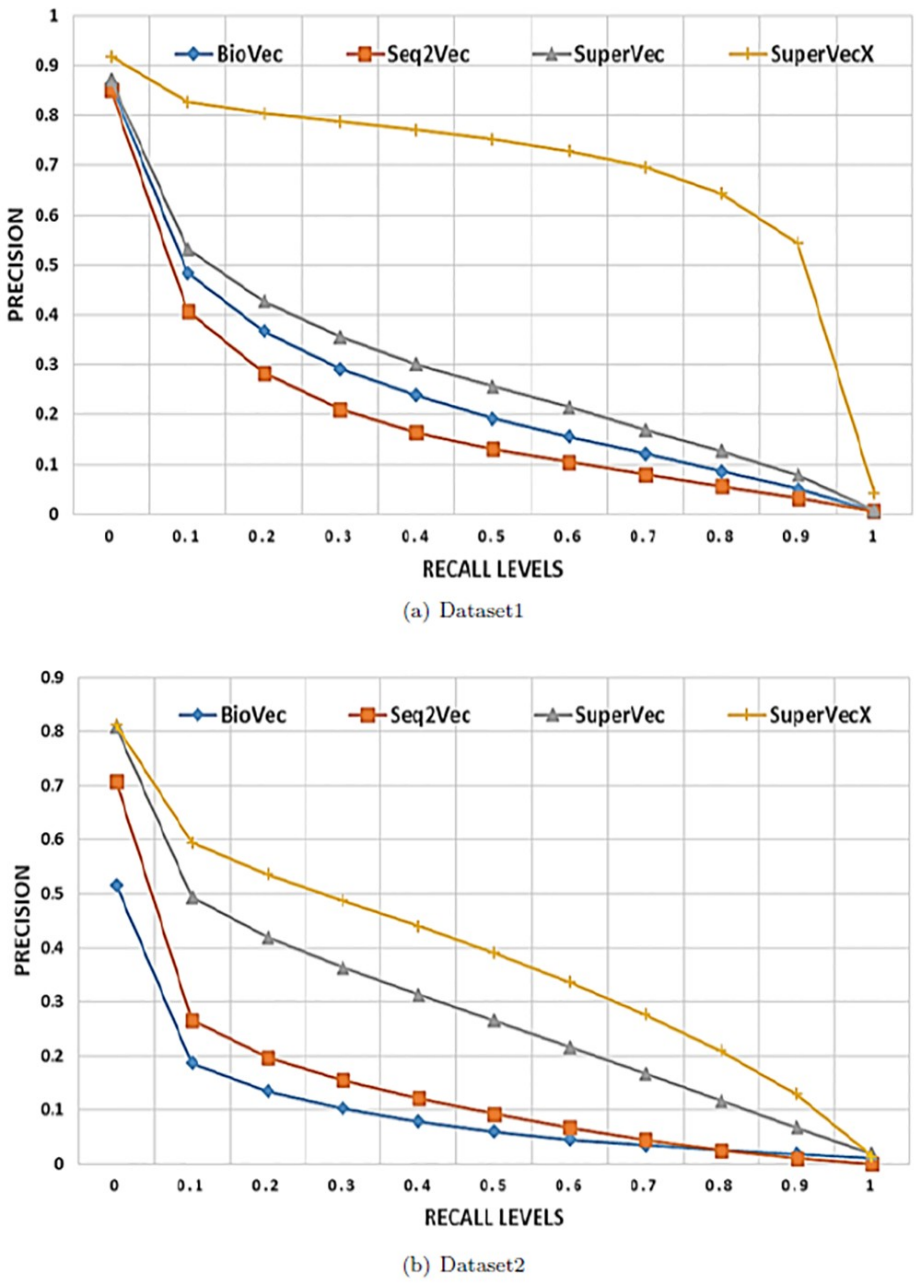
Supervised Vs unsupervised: Average interpolated precision values at 11 recall levels for dataset1 and dataset2. For dataset1 the results are averaged for ∼60*k* queries, the database size is ∼90*k* (200 classes); for dataset2 the database size is ∼58*k* sequences (100 classes) and the results are averaged over ∼38*k* queries.

These experiments are subsequently extended to much larger databases involving a large number of classes and sequences. We again follow experimental setup 1 and limit our analysis to the 200 classes (150,324 sequences) from dataset1, ensuring a minimum size of 400 samples. We also test our proposed methods on the largest 100 classes (96,612 sequences) of dataset2. This dataset is more challenging than dataset1 as, no two sequence shares more than 50% sequence similarity. The results of these experiments are provided in Fig 7.

Note that while we only provide results for the larger databases (200 and 100 classes) in Fig 7, we performed the same retrieval task on the databases of different sizes and find that the results remain consistent with the experiments shown.

Our observations may be summarized as follows:

- SuperVec and SuperVecX consistently outperform the baseline biological sequence embedding approaches on the sequence retrieval task for a wide range of database sizes.
- RepL methods are substantially faster than BLAST and provide a reduction up to ∼50*X* in querying time, albeit with some loss of precision for larger databases (refer Fig 12).
- When compared to the MMseqs2, RepL methods provide comparable querying time. Also, these methods provide better precision values than MMseqs2 for higher recall levels (refer Fig 12).
- The presence of a large number of sequences and classes in the database leads to deterioration in the performance of all methods. Although the retrieval performance of supervised methods also deteriorates for larger databases, it still consistently performs better than Seq2Vec and BioVec, most likely as a result of its ability to incorporate the class label information in the sequence embeddings.
- In general, SuperVec performs better than SuperVecX for small class databases, whereas SuperVecX outperforms SuperVec for larger database sizes. We further observe that SuperVec performance is similar for both datasets 1 and 2, whereas the performance of SueprVecX is seen to degrade for dataset2 as compared to dataset1.

**Robustness of SuperVec and SuperVecX**. To study the robustness of our approaches, we conduct experiments on larger databases, this time following the different setup discussed below.

- **Setup 2:** In this setup the sequences are chosen randomly from each class in the ratio 60:20:20, generating the training, database, and query set. Here the training sequences are used for training the models, whereas the database and query sets are reserved for validation on the retrieval task. Once the models are trained, the process followed to generate sequence embeddings and retrieval of homologous sequences for a given query is the same as described in setup 1. Note that this change has little effect on BioVec generated sequence embeddings as we use the *k-mer* embeddings directly from [7].
  As mentioned before, setup 1 uses database sequences and their labels as part of the training process, leading to the possibility of overfitting with respect to the database sets. This setup is intended to show the robustness of our approach, demonstrating that our method gives retrieval results similar to those obtained in setup 1 even when a different set of sampled sequences are used to train the models.

**Fig 8:**
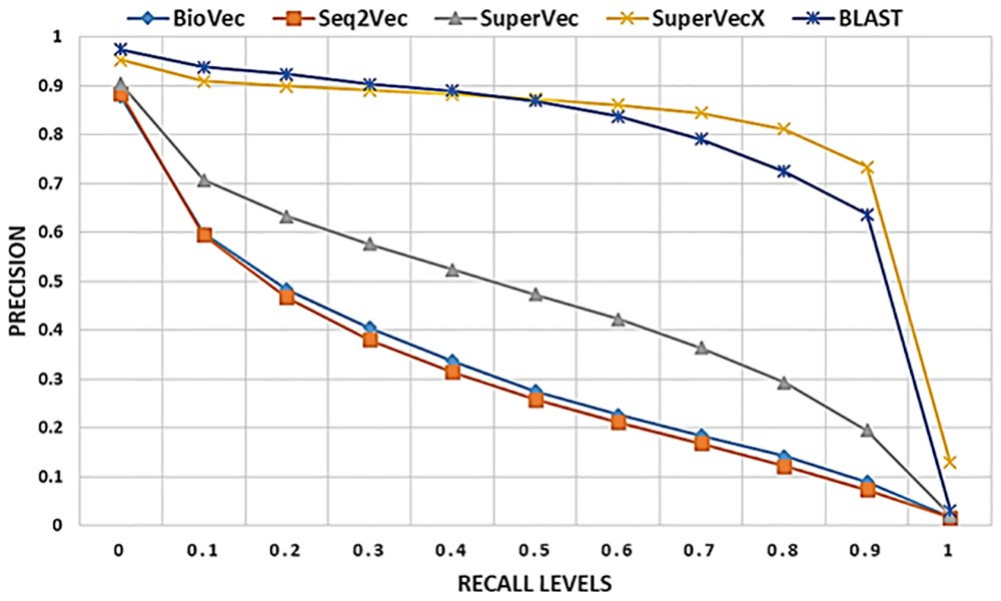
100 classes experiment: Comparison of interpolated average precision values for retrieval task performed on largest 100 classes database of dataset1 following setup 2.

The retrieval results obtained following setup 2 are consistent with setup 1, where supervised approaches outperform the baseline RepL methods. As noted before for the two-class experiment, SuperVecX is seen to outperform BLAST, especially at late recall levels. The retrieval result for the database of the largest 100 class of dataset1, having 75, 576 sequences, is provided in Fig 8. Since the database and training sequences are different in setup 2, the superior results obtained using SuperVec(X) suggest that SuperVec(X) transfers the information learned from the training sequence and their labels efficiently to the database and query sequence embeddings.

**Retrieval using hierarchical Approach: H-SuperVec(X)**. As discussed before, the hierarchical approach is specifically designed for the sequence retrieval task and may be used with any supervised RepL method. When used with our proposed methods—SuperVec or SuperVecX—we call these methods H-SuperVec and H-SuperVecX respectively. In this approach, the set of classes in the database is partitioned into a series of exclusive and exhaustive subsets leading to a binary tree (Fig 4). We then train SuperVec(X) model for each of the subset nodes of the tree. For a given set of classes, many such partitions are possible, and the choice of partitioning may affect the training of SuperVec(X) network, which in turn affects the query-subject similarity computation and subsequent retrieval performance.

To analyze the effect of partitions on retrieval results, we perform the retrieval experiment on different splits of a randomly chosen set of eight classes from the dataset1, with the experimental protocol following setup 1. The details are provided in S2 Appendix. The results from this experiment show that the choice of the partition at the root node has a negligible impact on the performance of H-SuperVec; in other words, a random partition may be chosen for applying H-SuperVec.

**Fig 9:**
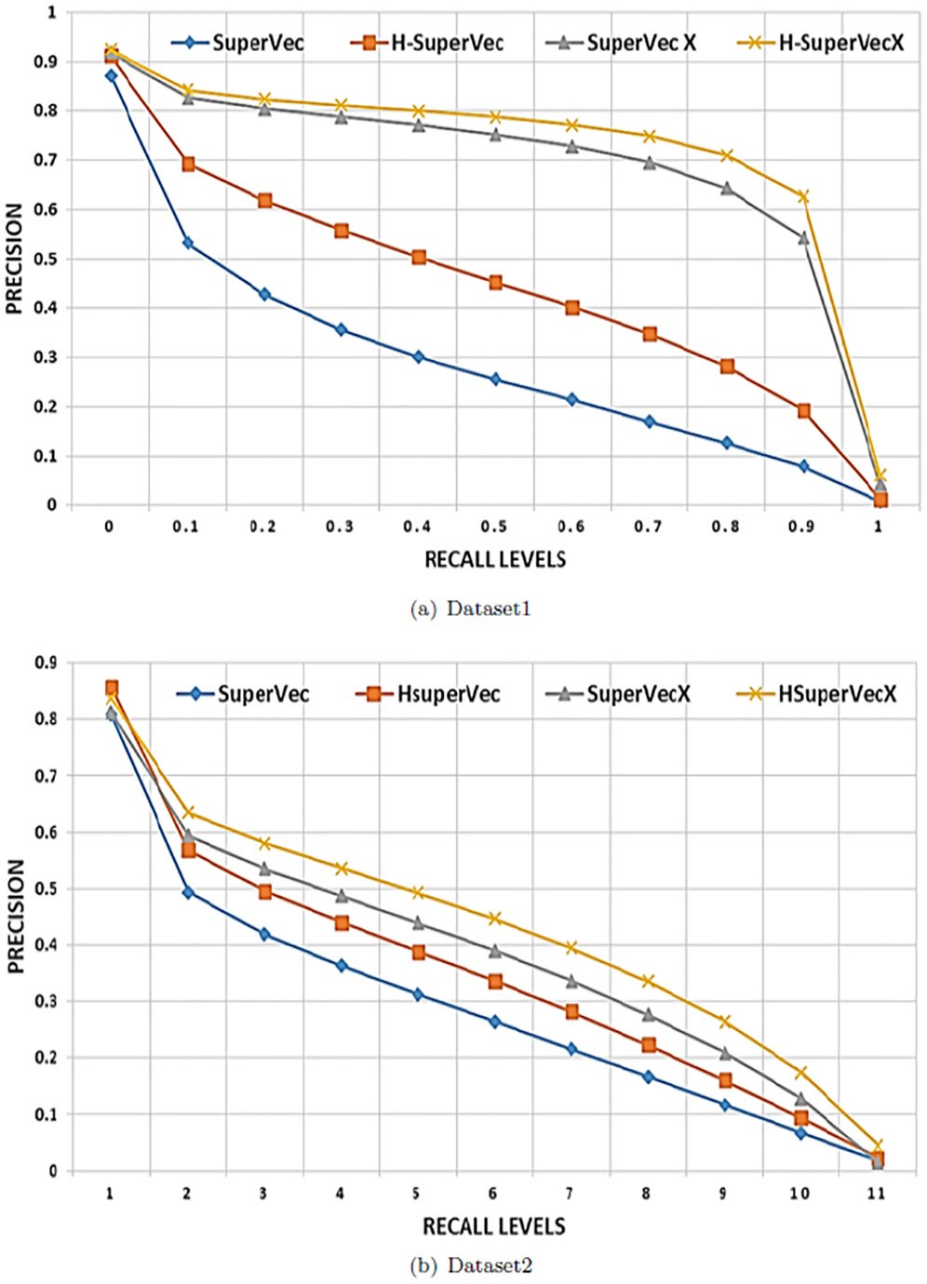
H-SuperVec(X) vs SuperVec(X): Retrieval performance comparison of the hierarchical and vanilla embedding approaches for dataset1 and dataset2.

Based on the observations from the eight class experiments, we use a random partition of equal size for applying the hierarchical approach on larger databases—(i) ∼90*k* sequences (largest 200 classes) of dataset1 and (ii) ∼58*k* sequences (largest 100 classes) of dataset2. It is observed that H-SuperVec provides an improvement in precision values as high as ∼70%, whereas H-SuperVecX provides improvement of ∼30% compared to SuperVec and SuperVecX respectively. The results are provided in Fig 9. These results validate our claim that using multiple observations of the distance gives a better measure of the similarity of the sequences in the embedding space, leading to superior performance compared to SuperVec or SuperVecX on the retrieval task.

We consider the querying time required for each of these methods in the section below.

**Querying time**. In this section, we provide a comparison of the querying time of all the considered methods for the sequence retrieval task. For the querying time computation, we use a database of ∼90*k* sequences and 200 classes of dataset1 that is searched for a set of 1108 queries. These query sequences are selected randomly and in equal proportion from each class. We report the querying time for two possible cases: (i) when all the queries are processed together; results are provided in Table 4, (ii) when the queries are provided sequentially to the system, i.e., one after another; results are provided in Table 5. Querying times are seen to be similar across the RepL methods; the slight variation among the querying time of these methods is observed because of the difference in embedding generation time. The nearest neighborhood search time remains the same for all of the RepL methods. Since the SuperVec(X) models required for H-SuperVec(X) are generated in parallel, the querying time for H-SuperVec(X) remains approximately same as for SuperVec(X).

**Table 4:** Querying time: Overall querying time for 1108 queries when processed together for the database of size 90*k* (200 classes) from dataset1.

Methods	BioVec	Seq2Vec	SuperVec(X)	H-SuperVec(X)	BLAST	MMseqs2
real time	0*m*40*sec*	0*m*15*sec*	0*m*15*sec*	0*m*21*sec*	13*m*43*sec*	0*m*23*sec*
sys+user time	0*m*54*sec*	0*m*36*sec*	0*m*36*sec*	0*m*50*sec*	13*m*43*sec*	4*m*55*sec*

**Table 5:** Querying time: Overall querying time for 1108 queries when processed serially for the database of size 90*k* (200 classes) from dataset1.

Methods	BioVec	Seq2Vec	SuperVec(X)	H-SuperVec(X)	BLAST	MMseqs2
real time	1m1*sec*	0*m*39*sec*	0*m*39*sec*	0*m*42*sec*	19*m*31*sec*	1*m*2*sec*

The RepL approaches provide a speed improvement up to ∼50*X* vis-a-vis BLAST. When compared to MMseqs2 (run on 16 threads), we note that the real-time is in the similar range as RepL methods, whereas the system+user time of MMseqs2 is ∼13*k* higher than the RepL methods. It shows that MMseqs2 is optimized to run on multiple threads that provide a gain in real-time. On analyzing the querying time results further, we note that querying a time for RepL methods reported in Table 4 is dominated by the embedding generation time (∼14*sec*) while the neighborhood search time is minimal (∼1*sec*). The overall querying time of the RepL method is thus can be further improved by optimizing the embedding generation code to run on multiple threads.

**Fig 10:**
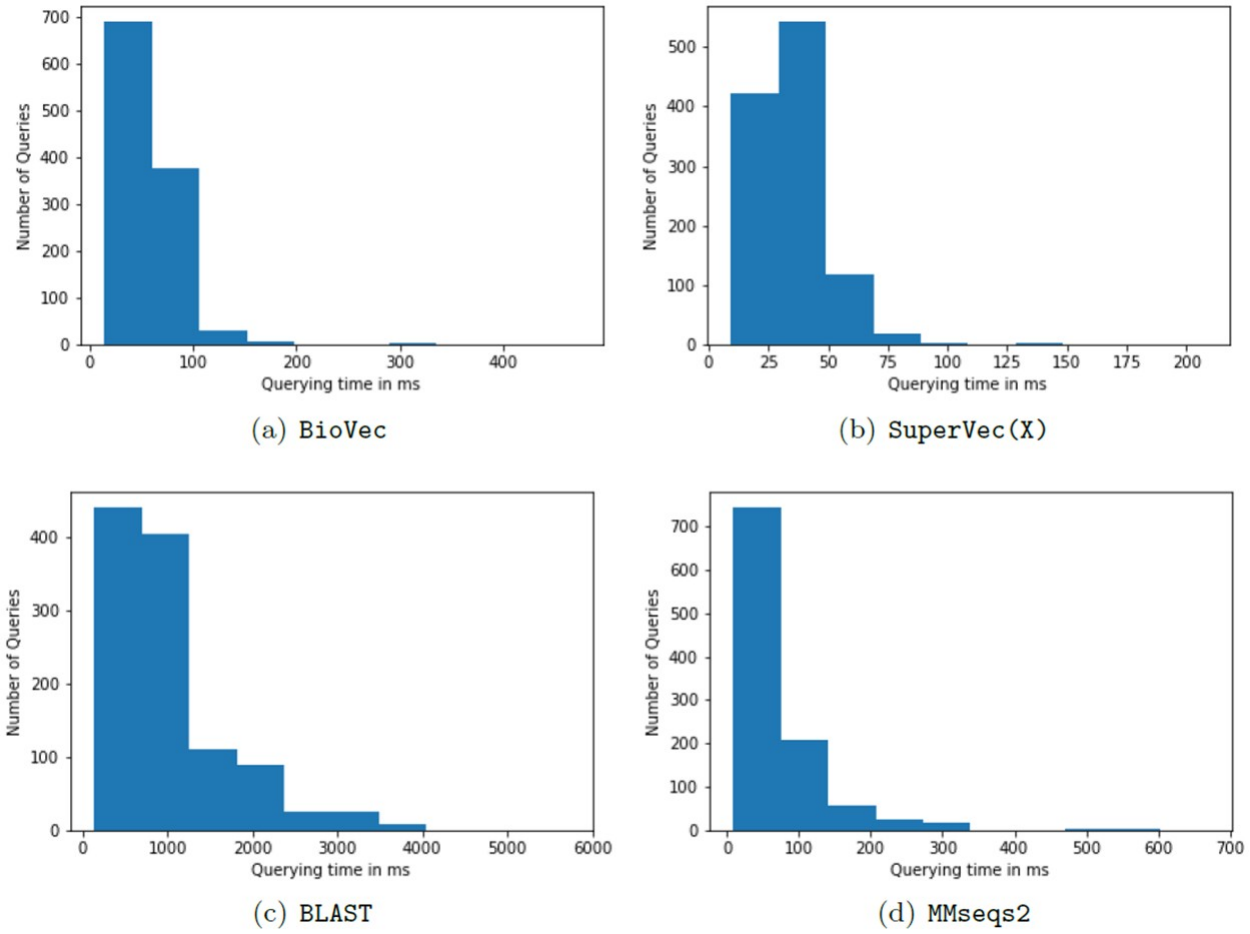
Querying time histogram: Querying time histogram for different methods.

We also note that the retrieval of similar sequences from a database for a query varies considerably based on the size and composition of the query and database, especially for alignment-based methods. Therefore, for further comparison of alignment and alignment-based methods we provide the distribution of BLAST, MMseqs2, BioVec and SuperVec(X) in Fig 10. The distribution of Seq2Vec and H-SuperVec(X) is noted to be similar to the SuperVec(X). As seen in Fig 10, the variance of SuperVec(X) is smallest among all; also, it performs better than other methods as the querying time for most of the queries in SuperVec(X) is limited to 50*msec*. In contrast, for BLAST, querying time is higher, and most of them are limited between 1 − 2*sec*. For MMseqs2 and BioVec, the distribution is almost similar, and most of the queries take less than 150*msec*.

The advantages offered by H-SuperVec(X)—the gain in processing time and the improved retrieval results when compared to the earlier models (SuperVec(X), BioVec, and Seq2Vec) —suggest that we can use it along with high precision method like BLAST for significantly faster retrieval of sequences with only a modest reduction in fidelity of the results. We now consider this hybrid approach in detail.

**Fig 11:**
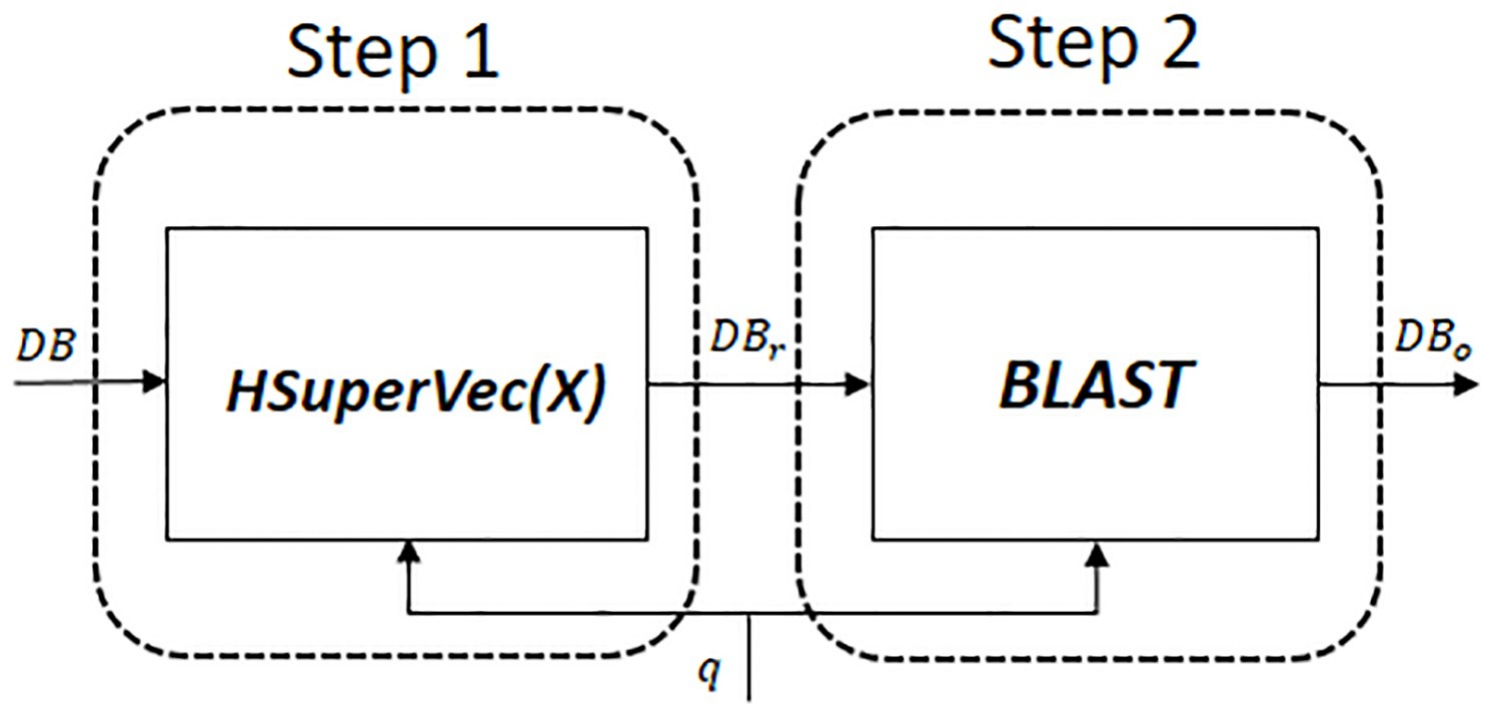
Hybrid approach: Step1 uses H-SuperVec(X) for pruning the original database (*DB*) and gives reduced database (*DB*_*r*_). In step 2, BLAST re-ranks *DB*_*r*_ based on alignment-based similarity between its sequences and the given query, *q* and finally provide the list of retrieved sequences, *DB*_*o*_.

**Hybrid approach: H-SuperVec(X)+BLAST**. The proposed hybrid approach—H-SuperVec(X)+BLAST (H(X)+BLAST) follows a two-step retrieval process. H-SuperVec(X) is utilized initially to prune the original database to produce a list of results potentially relevant for the given query. Here the selection is based on the nearest neighbor search in the database embedding space. The list of possible relevant subjects (the reduced database) obtained via H-SuperVec(X) is then provided to BLAST for re-ranking in accordance with the given query. Fig 11 shows the block diagram for the H(X)+BLAST approach, where *DB* represents the original database, *DB*_*r*_ the reduced database set obtained from the first step and *DB*_*o*_ the final ranked list of similar sequences for the given query.

The size of the *DB*_*r*_ is fixed by choosing a sufficient number of nearest neighbors (NN) for a given query. For our experiment, we keep NN = 15*k*, thereby allowing BLAST to operate on a comparatively small database (*DB*_*r*_). This provides a significant improvement in querying time compared to the direct application of BLAST to the original database. The average querying times using HSuperVec+BLAST and BLAST to process ∼60 *k* queries on a database of ∼ 90*k* sequences are ∼ 400*msec* and 1 *sec* respectively. Also, the use of BLAST in step 2 gives a performance improvement over H-SuperVec(X) on the retrieval task. With the increase in database sizes, we expect to see further improvements in querying time and retrieval performance of H(X)+BLAST as compared to BLAST and H-SuperVec(X) respectively. Utilizing both approaches together thus gives us the best of both worlds —faster processing of queries and higher precision levels in the results.

**Fig 12:**
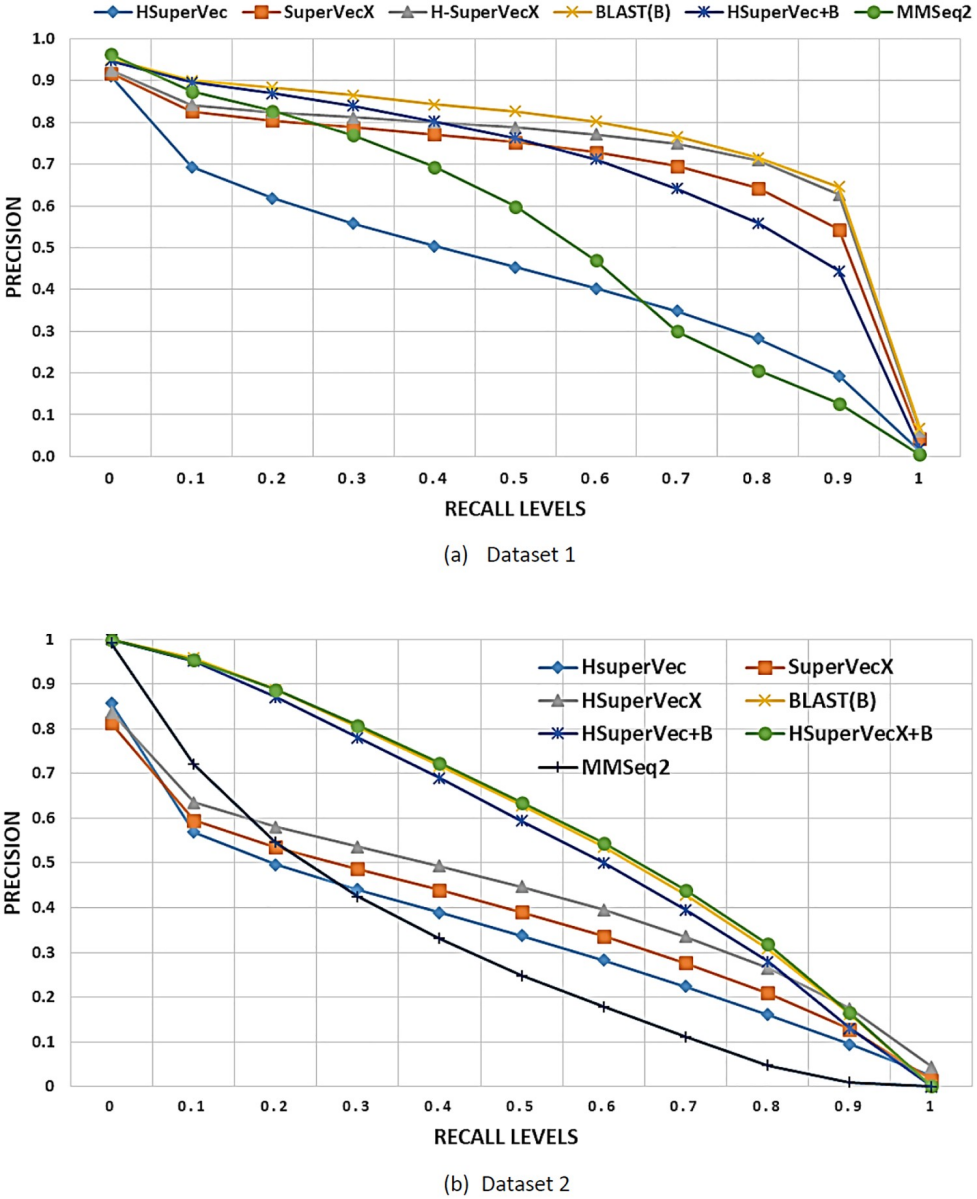
Retrieval result for hybrid approaches: In (a) The results are averaged over ∼ 60*k* queries on the database of ∼ 90*k* sequences and 200 classes. The AUPR values for the methods shown are as follows, HSuperVec: 0.451, SuperVecX: 0.703, H-SuperVecX: 0.742, BLAST: 0.776, HSuperVec+B: 0.701, MMseqs2: 0.535. In (b) the results are averaged over ∼ 38*k* queries on the database of ∼ 58*k* sequences and 100 classes. The AUPR values for the methods shown are as follows, HSuperVec: 0.343, SuperVecX: 0.381, HSuperVecX: 0.43, BLAST: 0.593, HSuperVec+B: 0.569, HSuVecX+B: 0.597 MMseqs2: 0.311.

Fig 12 provides a comparison of the hybrid approach with other embedding approaches, BLAST, and MMseqs2 on the retrieval task for databases from dataset1 and dataset2. As shown, a substantial improvement can be achieved with the hybrid approach over the other embedding based approaches and MMseqs2, with the results obtained comparable in precision to BLAST.

**Fig 13:**
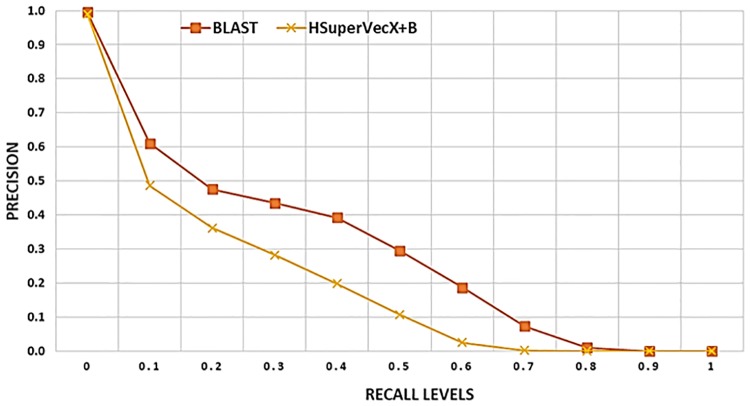
Retrieval performance comparison of hybrid approach and BLAST for database of size ∼650, 000 sequences (1886) classes from dataset1. The results are averaged over 800 queries randomly chosen from largest two classes.

For a much larger database of ∼650, 000 sequences (1886 classes) the results for BLAST and HSuperVecX+BLAST are provided in Fig 13. Since the querying time required by BLAST is large (∼1.42 per query), we show the result for 800 queries randomly chosen from the largest two classes.

As shown, we can overcome the gap between HSuperVecX and BLAST by using the hybrid approach even for this large database. The average querying time for this database comes out to be 0.36 sec for HSuperVecX+B and 1.42 sec for BLAST, thereby giving a speedup of 4.5× in querying time. Note that, for the hybrid approach, we chose the size of the reduced database to be 15*k*, increasing this size would further improve the performance at the cost of some increase in querying time.

**Fig 14:**
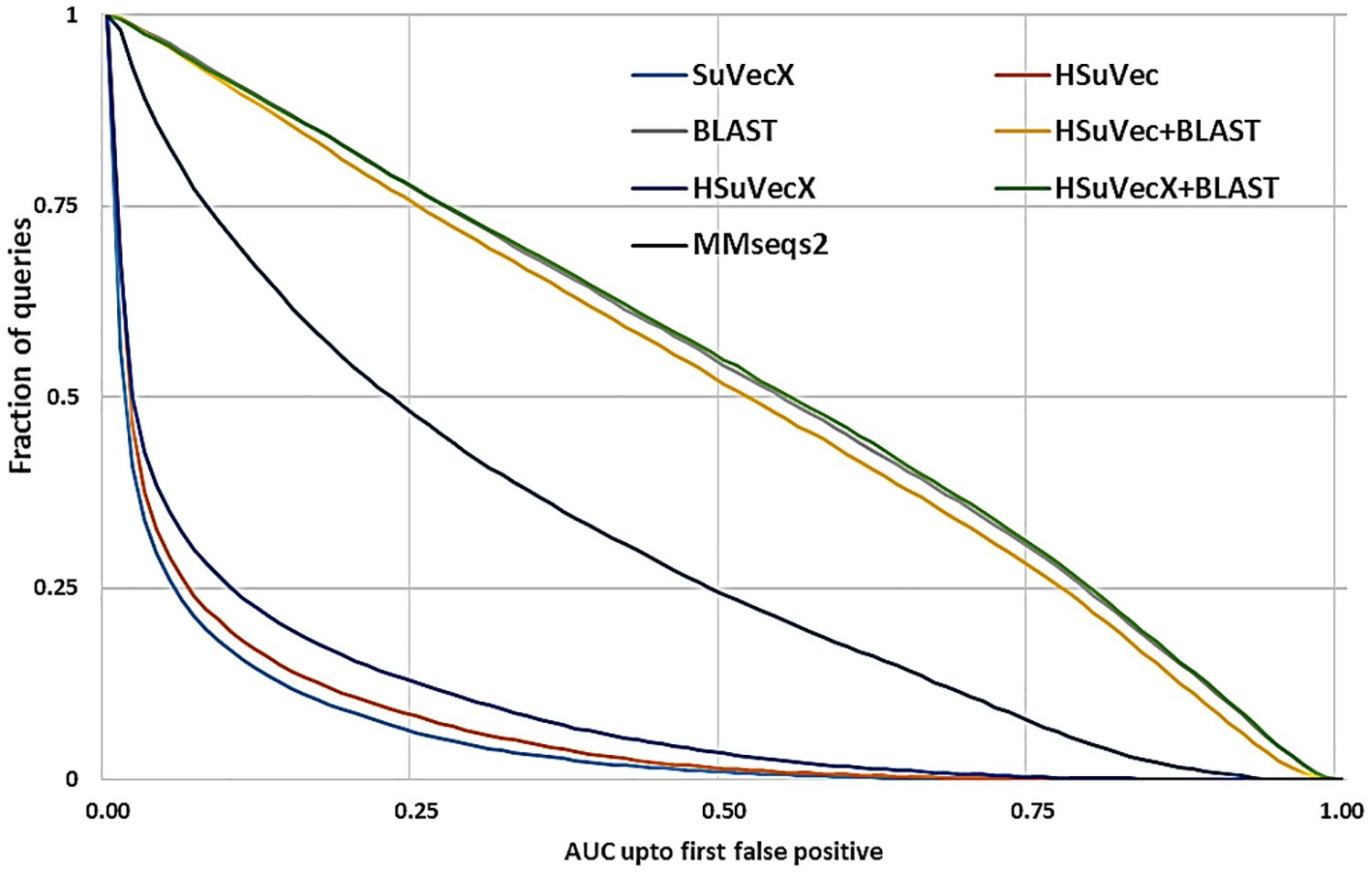
Sequence searching sensitivity assessment: Cumulative distribution of area under the curve (AUC) sensitivity for ∼ 38*k* queries on the database of ∼ 58*k* sequences and 100 classes of dataset2. Higher curves signify higher sensitivity.

Hybrid approaches are also expected to improve the sensitivity of RepL based methods. Here the sensitivity is defined in terms of the ability of a method to retrieve the maximum number of relevant sequences (true-positive) from the database before a non-relevant (false-positive) is extracted. This sensitivity is termed *AUC1*, which is defined as the fraction of true positive sequences retrieved prior to the appearance of the first false positive [5]. A comparison of the cumulative distribution of *AUC1* values for different methods is provided in Fig 14, here the higher curve signifies higher sensitivity of the method. As shown, the hybrid approach (HSuperVecX+B) significantly improves the sensitivity of HSuperVecX and moves the curve close to BLAST.

Furthermore, there is some scope for further improvement in methodologies. Improved RepL approaches may be expected to yield better database and query embeddings, so that the relevant subjects for a given query may be confined to a close neighborhood in the database embedding space. This would be likely to lead to improved performance on the retrieval task. Improvements in RepL approaches would also allow us to choose a smaller number of candidate relevant subjects for a given query, thus providing further gains in processing time and performance for hybrid approaches.

### 5.2 Protein classification tasks

In addition to the sequence retrieval task, we demonstrate the effectiveness of SuperVec and SuperVecX generated embeddings for (i) Toxin prediction (ii) Sub-cellular localization and (iii) enzyme classification tasks. Here Toxin and enzyme prediction tasks are binary classification problems, whereas the subcellular localization is a multi-class (4-class) classification problem.

#### 5.2.1 Data

We use the toxin prediction dataset provided by ToxinClassifier [19]. It contains 8093 protein sequences annotated as animal toxins and venoms. For the negative set, we pick the sequences from the ‘Hard setting’ provided in the ToxinClassifier. It contains 7043 protein sequences.

For sub-cellular localization we use the TargetP 4-class dataset [20]. This dataset contains (i) 371 mitochondrial (ii) 715 pathway (iii) 1214 nuclear and (iv) 438 cytosolic protein sequences.

For the enzyme prediction problem, we pick the dataset provided by the ‘NEW’ dataset of Deepre [21]. It contains 22,168 protein sequences for the enzyme and non-enzyme classes.

#### 5.2.2 Experimental design and evaluation

The classification results are computed for a 5-fold cross-validation setup. We report macro-precision, recall, and F1 score as the evaluation metric. The macro averaging is done by computing the metrics for each class and finally averaging them. Such averaging gives equal importance to all the classes. The macro F1 computes the geometric mean of precision and recall values and hence captures the trade-off between them.

For classification, we use a Multi-layer Perceptron (MLP) classifier with 3 hidden layers (70,50,20), with Rectified Linear Unit (ReLU) as the nonlinear activation function. At the output of MLP, the softmax function is used to compute the posterior probabilities associated with each class. For comparison, we use the results provided by [8] where the authors perform same classification tasks and reported the results for the baseline unsupervised representations—k-mer, BioVec, ProtVecX and their combinations.

#### 5.2.3 Results and discussion

Protein classification results using MLP classifier on toxin prediction, sub-cellular localization, and enzyme prediction is provided in Table 6. For sub-cellular location and enzyme prediction tasks, SuperVec(X) outperforms unsupervised embeddings, ProtVec and ProtVecX. For toxin prediction task, SuperVecX perform at par with other embeddings but surprisingly SuperVec do not perform better than others, which needs to be further investigated.

**Table 6:** Classification results: Comparing SuperVec, SuperVecX and other embeddings on various classification tasks.

Dataset	Representation	5-fold cross validation results		
		macro-precision	macro-recall	macro-F1
Toxin prediction	3-mer	0.89	0.89	0.89
	ProtVec	0.88	0.88	0.88
	ProtVecX	0.88	0.88	0.88
	3-mer+ProtVec	0.90	0.89	0.89
	3-mer+ProtVecX	0.90	0.90	0.90
	SuperVec	0.86 ± 0.01	0.86 ± 0.01	0.86 ± 0.01
	SuperVecX	0.90 ± 0.01	0.90 ± 0.01	0.90 ± 0.01
Subcellular location prediction	3-mer	0.65	0.59	0.60
	ProtVec	0.60	0.57	0.58
	ProtVecX	0.57	0.57	0.57
	3-mer+ProtVec	0.68	0.60	0.62
	3-mer+ProtVecX	0.66	0.60	0.61
	SuperVec	0.63 ± 0.02	0.60 ± 0.03	0.61 ± 0.02
	SuperVecX	0.61 ± 0.01	0.57 ± 0.02	0.58 ± 0.01
Enzyme prediction	3-mer	0.70	0.73	0.71
	ProtVec	0.68	0.70	0.69
	ProtVecX	0.69	0.71	0.70
	3-mer+ProtVec	0.70	0.73	0.71
	3-mer+ProtVecX	0.71	0.73	0.72
	SuperVec	0.74 ± 0.001	0.74± 0.001	0.74± 0.001
	SuperVecX	0.73± 0.001	0.73± 0.002	0.73± 0.001

The results reported in Table 6 for unsupervised embeddings i.e. 3-mer, ProtVec, ProtVecX and their combination are directly obtained from [8]; the size of these embeddings is 8000, 500 and 500 respectively, whereas the SuperVec and SuperVecX embeddings are of dimension 100. Note that for any specific classification task, the SuperVec(X) models are trained on the protein sequences available for that task. The better performance of supervised embeddings on classification tasks shows that the information present in the sequences can be well captured in a low dimensional vector. As noted before, SuperVec relies on contextual and class information whereas SuperVecX utilizes only class labels. The difference in the performance of SuperVec and SuperVecX over these tasks suggests that for some tasks, class label information may be sufficient, but for some tasks, contextual information may further improve the embeddings.

## 6 Conclusions

This paper introduces two new supervised approaches—SuperVec and SuperVecX for learning embeddings of biological sequences. The key observation is that the knowledge of sequence labels leads to more meaningful embeddings that can be utilised for various downstream bioinformatics tasks using growing machinery of machine learning techniques. Not only are the proposed embedding models computationally inexpensive to train, but even the application of these models is also sufficiently fast. This makes such embedding based strategies suitable for the processing of huge sequence databases. Using a combination of these models, as demonstrated by their Hierarchical versions H-SuperVec(X), further improves the performance for the database retrieval task. Given that the embedding models do not employ sequence alignment algorithms directly, there is a great scope of hybrid frameworks that use both alignment and non-alignment models to better represent and process the biological sequences. Building on such hybrid approaches is a potentially useful direction of research.

## Supporting information

S1 AppendixThe derivation of update equations for SuperVec parameters−s_*i*_, $k_{i_{j}}$, g_*q*_, s_*r*_ and s_*z*_.(PDF)

S2 Appendix The eight class experiment conducted for analyzing the effect of split on retrieval performance of hierarchical approaches. (PDF)

S3 Appendix The comparison of percentage improvement in precision value at 60% recall for all methods on dataset1. (PDF)

S4 Appendix Detailed description of Dataset1 and dataset2. (PDF)

S1 Fig t-SNE plots. (PDF)
