# Supplementary material for: Learning supervised embeddings for large scale sequence comparisons
Source: PLoS One. 2020 Mar 13;15(3):e0216636. doi: 10.1371/journal.pone.0216636 (PMC7069636; doi:10.1371/journal.pone.0216636)
Supplement: S1 Appendix — (PDF) [file pone.0216636.s001.pdf]

Update Equations

The update equations for the parameters - $\mathbf{s}_i$, $\mathbf{k}_{i_j}$, $\mathbf{g}_q$, $\mathbf{s}_r$ and $\mathbf{s}_z$ of `SuperVec` are obtained using gradient descent. As usual, parameter updates rely on their gradient with respect to the objective function. If we want to find the value of $\mathbf{x}$ for which f($\mathbf{x}$) is minimised, we initialize $\mathbf{x}$ randomly and update its value in each iteration. The update equation for $\mathbf{x}$ is then

$$\mathbf{x}^{new} = \mathbf{x} - \eta \frac{\partial \mathrm{f}(\mathbf{x})}{\partial \mathbf{x}}, \tag{1}$$

where η is the learning rate. For `SuperVec` the overall loss function is given by

$$\mathrm{J}(\mathbf{S}, \mathbf{K}) = \sum_{s_i \in \mathcal{S}} \sum_{j=1}^{n_i} \left[\underbrace{- \log \ \mathrm{P}r \left[k_{i_j} \mid C_{i_j}, \ s_i \right]}_{NN1} - \gamma \sum_{z \in \mathcal{I}_i^+} \underbrace{\log \ \mathrm{P}r \left[s_z \mid s_i \right]}_{NN2} \right]. \tag{2}$$

For $NN1$ we employ a hierarchical softmax approach and for $NN2$ we use a negative sampling technique. Hierarchical softmax (HS) is based on a binary tree ($\mathcal{T}$) construction, where each leaf maps to a *k-mer* in $\mathcal{K}$; also, since it is a binary tree, any leaf (*k-mer*) can be reached rapidly from the root by traversing a unique path. We denote the qth node in such a path from the root to the leaf *k-mer* k_{i_j} as $n(k_{i_j}, q)$ and the corresponding vector as $\mathbf{g}_{k_{i_j},q}$. Considering a stack of these internal node vectors of $\mathcal{T}$ yields the internal node matrix, $\mathbf{G}$. The probability for a specific *k-mer* k_{i_j} is obtained by traversing from the root to the leaf corresponding to k_{i_j} and multiplying the contributions (defined below) from each internal node in the path. When calculated using the hierarchical softmax approach, $\mathrm{P}r \left[k_{i_j} \mid C_{i_j}, \ s_i \right]$ may then be written

$$\prod_{q=1}^{L_{i_j}-1} \sigma \left(child(q-1) \cdot \langle \mathbf{g}_{k_{i_j},q}, \mathbf{h}_{i_j} \rangle \right), \tag{3}$$

where $child(q-1) = 1$ if $n(k_{i_j}, q)$ is the left child of $n(k_{i_j}, q-1)$ and -1 otherwise, and L_{i_j} is the length of the path i.e. the total number of nodes in the path from the root to the jth *k-mer* of sequence s_i; $\mathbf{h}_{i_j}$ is the sum of vectors corresponding to context C_{i_j} and sequence s_i. Finally, the overall objective function is written as,

$$\begin{aligned} \mathrm{J}^{'}(\mathbf{S}, \mathbf{K}, \mathbf{G}) = \min \sum_{s_i \in \mathcal{S}} \sum_{j=1}^{n_i} \sum_{q=1}^{L_{i_j}-1} \Bigg[& - \log \ \sigma \left(child(q-1) \cdot \langle \mathbf{g}_{k_{i_j},q}, \mathbf{h}_{i_j} \rangle \right) \\ & - \gamma \sum_{z \in \mathcal{I}_i^+} \left(\log \sigma \left(\langle \mathbf{s}_z, \mathbf{s}_i \rangle \right) + \sum_{r \in \mathcal{I}_i^-} \log \sigma \left(- \langle \mathbf{s}_r, \mathbf{s}_i \rangle \right) \right) \Bigg]. \end{aligned} \tag{4}$$

The update equation for `SuperVec` parameters are obtained by differentiating Eq (4) w.r.t the parameter and takes the same form as given for $\mathbf{x}$ in Eq (1). For example, the update equation for parameter $\mathbf{s}_i$ is given as:

$$\mathbf{s}_i^{new} = \mathbf{s}_i - \eta \frac{\partial \mathrm{J}^{'}(\mathbf{S},\mathbf{K},\mathbf{G})}{\partial \mathbf{s}(i)}. \tag{5}$$

For notational convenience, we write $\mathrm{J}^{'}(\mathbf{S},\mathbf{K},\mathbf{G})$, $\mathbf{g}_{k_{i_j},q}$, $\mathbf{h}_{i_j}$, $child(q-1)$ as $\mathrm{J}^{'}$, $\mathbf{g}_q$, $\mathbf{h}$ and $[\![q-1]\!]$ respectively. The derivation of gradient of parameters w.r.t $\mathrm{J}^{'}$ are given below:

$$\frac{\partial \mathrm{J}^{'}}{\partial \mathbf{s}_i} = \sum_{q=1}^{L_{i_j}-1} \Bigg[\frac{-\partial \log\ \sigma([\![q-1]\!]\cdot\langle \mathbf{g}_q,\mathbf{h}\rangle)}{\partial \langle \mathbf{g}_q,\mathbf{h}\rangle} \cdot \frac{\partial \langle \mathbf{g}_q,\mathbf{h}\rangle}{\partial \mathbf{s}_i}$$
$$-\gamma \sum_{z\in\mathcal{I}_i^+} \left(\frac{\partial \log\sigma\left(\langle \mathbf{s}_z,\mathbf{s}_i\rangle\right)}{\partial \langle \mathbf{s}_z,\mathbf{s}_i\rangle} \cdot \frac{\partial \langle \mathbf{s}_z,\mathbf{s}_i\rangle}{\partial \mathbf{s}_i} + \sum_{r\in\mathcal{I}_i^-} \frac{\partial \log\sigma\left(-\langle \mathbf{s}_r,\mathbf{s}_i\rangle\right)}{\partial\left(-\langle \mathbf{s}_r,\mathbf{s}_i\rangle\right)} \cdot \frac{\partial(-\langle \mathbf{s}_r,\mathbf{s}_i\rangle)}{\partial \mathbf{s}_i} \right) \Bigg]. \tag{6}$$

using the identities:

$$\frac{\partial \log\sigma\left(\mathbf{x}\right)}{\partial\left(\mathbf{x}\right)} = \sigma\left(-\mathbf{x}\right)$$
$$\frac{\partial \log\sigma\left(\text{-}\mathbf{x}\right)}{\partial\left(\text{-}\mathbf{x}\right)} = \sigma\left(\mathbf{x}\right) \tag{7}$$

$\frac{\partial \mathrm{J}^{'}}{\partial \mathbf{s}_i}$ is simplified as:

$$\frac{\partial \mathrm{J}^{'}}{\partial \mathbf{s}_i} = -\sum_{q=1}^{L_{i_j}-1} \Bigg[\sigma(-[\![q-1]\!]\cdot\langle \mathbf{g}_q,\mathbf{h}\rangle)[\![q-1]\!]\cdot\mathbf{g}_q$$
$$+\gamma \sum_{z\in\mathcal{I}_i^+} \left(\sigma\left(-\langle \mathbf{s}_z,\mathbf{s}_i\rangle\right)\cdot\mathbf{s}_z + \sum_{r\in\mathcal{I}_i^-} -\sigma\left(\langle \mathbf{s}_r,\mathbf{s}_i\rangle\right)\cdot\mathbf{s}_r \right) \Bigg]. \tag{8}$$

further, using the identity:

$$\sigma\left(-\mathbf{x}\right) = 1 - \sigma\left(\mathbf{x}\right) \tag{9}$$

we can write first part of Eq (8) as,

$$\left[\sigma(-[\![q-1]\!]\cdot\langle \mathbf{g}_q,\mathbf{h}\rangle)\right][\![q-1]\!] = \left[1-\sigma([\![q-1]\!]\cdot\langle \mathbf{g}_q,\mathbf{h}\rangle)\right][\![q-1]\!]. \tag{10}$$

As noted before, $[\![q-1]\!]$ takes value 1 or -1. Eq (10) can be further simplified as:

$$\left[1-\sigma([\![q-1]\!]\cdot\langle \mathbf{g}_q,\mathbf{h}\rangle)\right][\![q-1]\!] = \begin{cases} 1-\sigma\left(\langle \mathbf{g}_q,\mathbf{h}\rangle\right) & if\ [\![q-1]\!]=1, \\ -\sigma\left(\langle \mathbf{g}_q,\mathbf{h}\rangle\right) & if\ [\![q-1]\!]=-1 \end{cases}$$
$$= t - \left(\sigma\left(\langle \mathbf{g}_q,\mathbf{h}\rangle\right)\right) \tag{11}$$

where $t = 1$ if $[\![q-1]\!] = 1$ and 0 otherwise.

Using Eq (11), we write the final expression for $\frac{\partial \mathrm{J}'}{\partial \mathbf{s}_i}$ as,

$$\frac{\partial \mathrm{J}'}{\partial \mathbf{s}_i} = -\sum_{q=1}^{L_{i_j}-1} \Bigg[t - \left(\sigma\left(\langle \mathbf{g}_q, \mathbf{h}\rangle\right)\right) + \gamma \sum_{z \in \mathcal{I}_i^+} \left(\sigma\left(-\langle \mathbf{s}_z, \mathbf{s}_i\rangle\right) \cdot \mathbf{s}_z + \sum_{r \in \mathcal{I}_i^-} -\sigma\left(\langle \mathbf{s}_r, \mathbf{s}_i\rangle\right) \cdot \mathbf{s}_r \right) \Bigg]. \quad (12)$$

Using Eq (8) and Eq (11) the gradients for the parameters: $\mathbf{k}_{i_j}$, $\mathbf{g}_q$, $\mathbf{s}_z$, $\mathbf{s}_r$ is given as below.

$$\begin{aligned} \frac{\partial \mathrm{J}'}{\partial \mathbf{k}_{i_j}} &= \sum_{q=1}^{L_{i_j}-1} \frac{-\partial \log\ \sigma([\![q-1]\!] \cdot \langle \mathbf{g}_q, \mathbf{h}\rangle)}{\partial \langle \mathbf{g}_q, \mathbf{h}\rangle} \cdot \frac{\partial \langle \mathbf{g}_q, \mathbf{h}\rangle}{\partial \mathbf{k}_{i_j}} \\ &= -\sum_{q=1}^{L_{i_j}-1} t - \sigma(\langle \mathbf{g}_q, \mathbf{h}\rangle) \cdot \mathbf{g}_q \end{aligned} \quad (13)$$

$$\begin{aligned} \frac{\partial \mathrm{J}'}{\partial \mathbf{g}_q} &= \sum_{q=1}^{L_{i_j}-1} \frac{-\partial \log\ \sigma([\![q-1]\!] \cdot \langle \mathbf{g}_q, \mathbf{h}\rangle)}{\partial \langle \mathbf{g}_q, \mathbf{h}\rangle} \cdot \frac{\partial \langle \mathbf{g}_q, \mathbf{h}\rangle}{\partial \mathbf{g}_q} \\ &= -\sum_{q=1}^{L_{i_j}-1} t - \sigma(\langle \mathbf{g}_q, \mathbf{h}\rangle) \cdot \mathbf{h} \end{aligned} \quad (14)$$

$$\begin{aligned} \frac{\partial \mathrm{J}'}{\partial \mathbf{s}_z} &= -\gamma \frac{\partial \log \sigma\left(\langle \mathbf{s}_z, \mathbf{s}_i\rangle\right)}{\partial \langle \mathbf{s}_z, \mathbf{s}_i\rangle} \cdot \frac{\partial \langle \mathbf{s}_z, \mathbf{s}_i\rangle}{\partial \mathbf{s}_z} \\ &= -\gamma \sigma\left(-\langle \mathbf{s}_z, \mathbf{s}_i\rangle\right) \cdot \mathbf{s}_i \end{aligned} \quad (15)$$

$$\begin{aligned} \frac{\partial \mathrm{J}'}{\partial \mathbf{s}_r} &= -\gamma \sum_{z \in \mathcal{I}_i^+} \frac{\partial \log \sigma\left(-\langle \mathbf{s}_r, \mathbf{s}_i\rangle\right)}{\partial\left(-\langle \mathbf{s}_r, \mathbf{s}_i\rangle\right)} \cdot \frac{\partial(-\langle \mathbf{s}_r, \mathbf{s}_i\rangle)}{\partial \mathbf{s}_r} \\ &= \gamma \sum_{z \in \mathcal{I}_i^+} \sigma\left(\langle \mathbf{s}_r, \mathbf{s}_i\rangle\right) \cdot \mathbf{s}_i \end{aligned} \quad (16)$$
