# Supplementary material for: Learning supervised embeddings for large scale sequence comparisons
Source: PLoS One. 2020 Mar 13;15(3):e0216636. doi: 10.1371/journal.pone.0216636 (PMC7069636; doi:10.1371/journal.pone.0216636)
Supplement: S2 Appendix — (PDF) [file pone.0216636.s002.pdf]

Herierchical Split Selection

In this experiment, we conduct a retrieval task on randomly selected database (of eight classes) from dataset1 to analyze the effect of partition on `H-SuperVec` performance. We compare the precision-recall values obtained by employing `H-SuperVec` against `SuperVec` and report the percentage change in the precision value at different recall levels. The results obtained from all 35 equal sized partitions of eight classes show a similar trend. The results for five of these partitioned is provided in Fig 1. These results demonstrate that:

- The choice of the partition at the root node has a negligible impact on the performance of `H-SuperVec`; in other words, a random partition may be chosen for applying `H-SuperVec`.

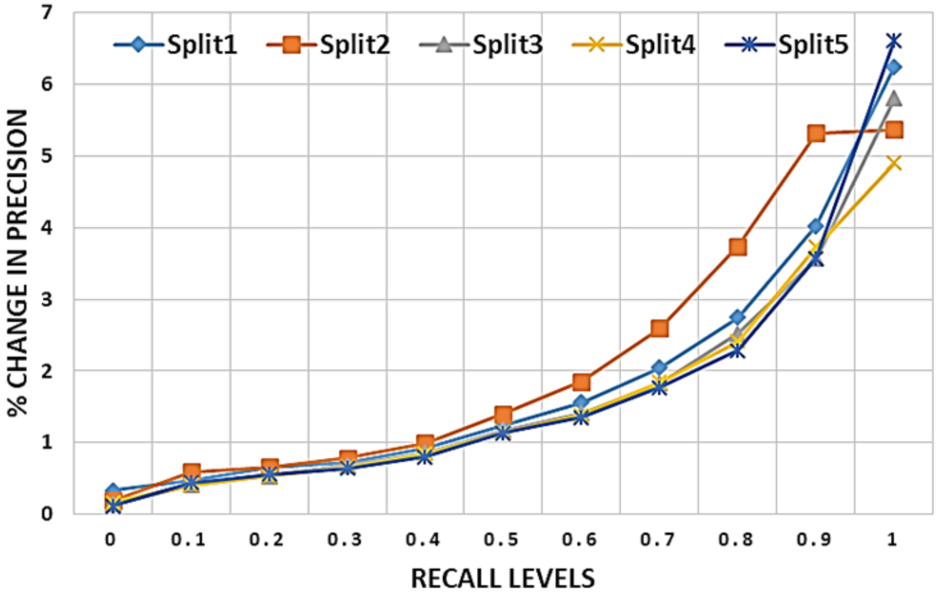

Fig 1. Percentage change in precision values: These plots shows the percentage change in precision values obtained for herierchical approach (`H-SuperVec`) as compared to `SuperVec` for the retrieval task performed on randomly chosen eight classes. The graph is shown for five splits among possible 35 splits.
