# Supplementary material for: Learning supervised embeddings for large scale sequence comparisons
Source: PLoS One. 2020 Mar 13;15(3):e0216636. doi: 10.1371/journal.pone.0216636 (PMC7069636; doi:10.1371/journal.pone.0216636)
Supplement: S3 Appendix — (PDF) [file pone.0216636.s003.pdf]

Comparison of all methods on retrieval task

In Fig 1, we provide the comparison of all methods on retrieval task on a database of $\sim 90k$ sequences; the results are averaged over $\sim 60k$ queries. As shown, the proposed supervised approaches provide a high gain in precision values over unsupervised methods and comparable performance as compared to BLAST.

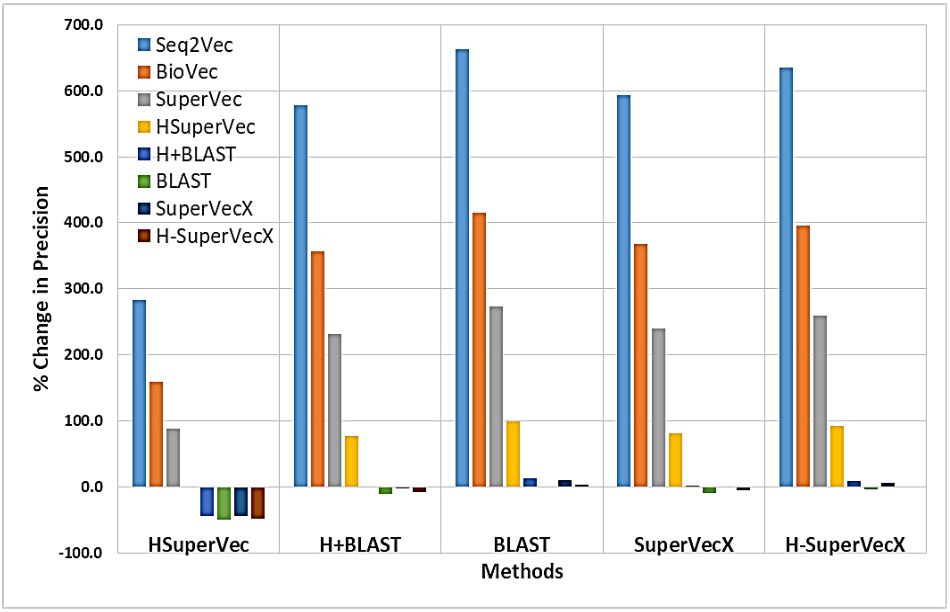

Fig 1. Improvement in precision: The plot shows the percentage improvement in precision value achieved by `SuperVec`, `HSuperVec(X)` and `H+BLAST` over other methods. Precision is compared at 0.6 recall for the database of $90k$ sequences and 200 classes from dataset1.
