# Supplementary material for: Learning supervised embeddings for large scale sequence comparisons
Source: PLoS One. 2020 Mar 13;15(3):e0216636. doi: 10.1371/journal.pone.0216636 (PMC7069636; doi:10.1371/journal.pone.0216636)
Supplement: S4 Appendix — (PDF) [file pone.0216636.s004.pdf]

Datset1

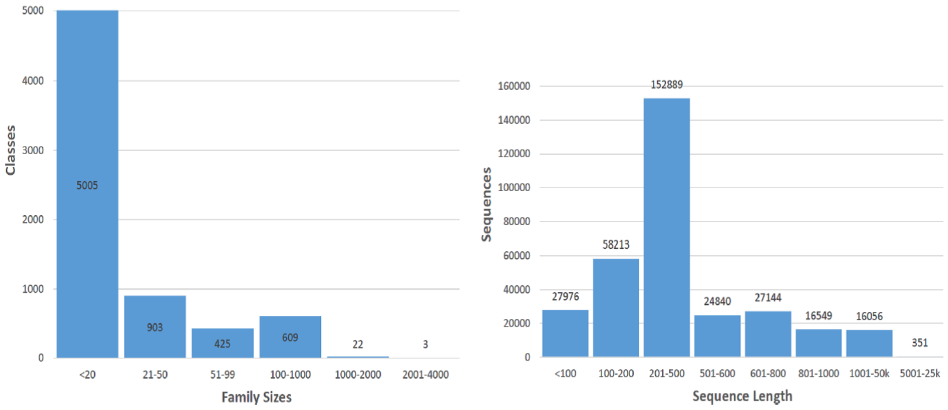

(a) Class size distribution (b) Sequence length distribution

Fig 1. Distribution of class sizes and lengths of sequences present in the complete dataset.

Dataset2

In total, there are 33 million representative sequences. For many of these sequences, no PFAM annotation is available. For our purposes, we keep only those sequences that are annotated with single “PFAM” entry, leaving us with 16968 unique “PFAM” entries (classes) containing 87, 358, 58 sequences. These classes vary in size, the largest one contains 81, 739, whereas more than 24, 00 classes have less than 10 entries. For experiments, we have to select classes that have a reasonable size to make training and test split we choose the classes that contain $400 - 1000$ sequences. There are 1866 such classes with a total of 11, 921, 50 sequences that comprises dataset2.
