# Supplementary figures and images for: Learning supervised embeddings for large scale sequence comparisons
Source: PLoS One. 2020 Mar 13;15(3):e0216636. doi: 10.1371/journal.pone.0216636 (PMC7069636; doi:10.1371/journal.pone.0216636)

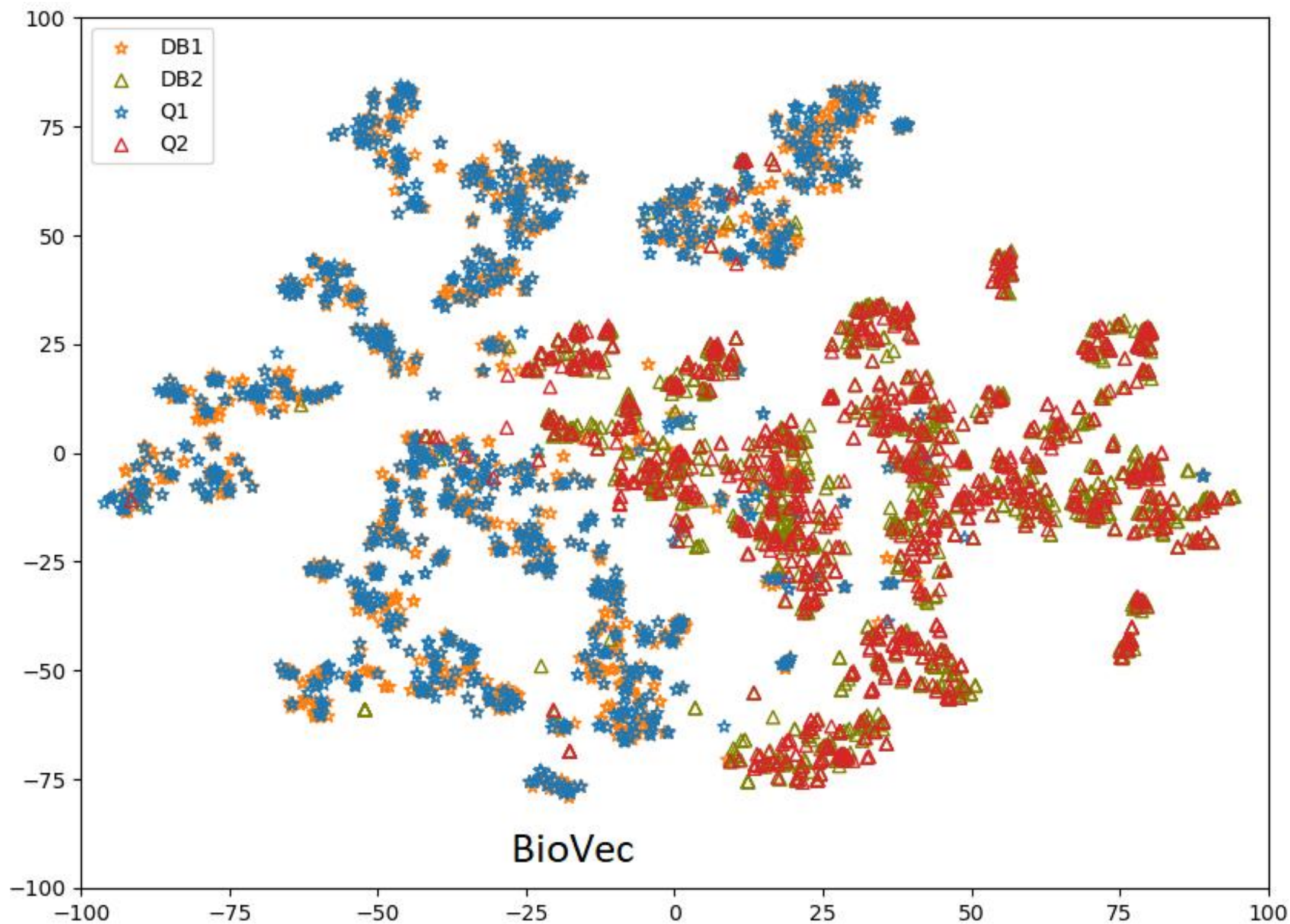

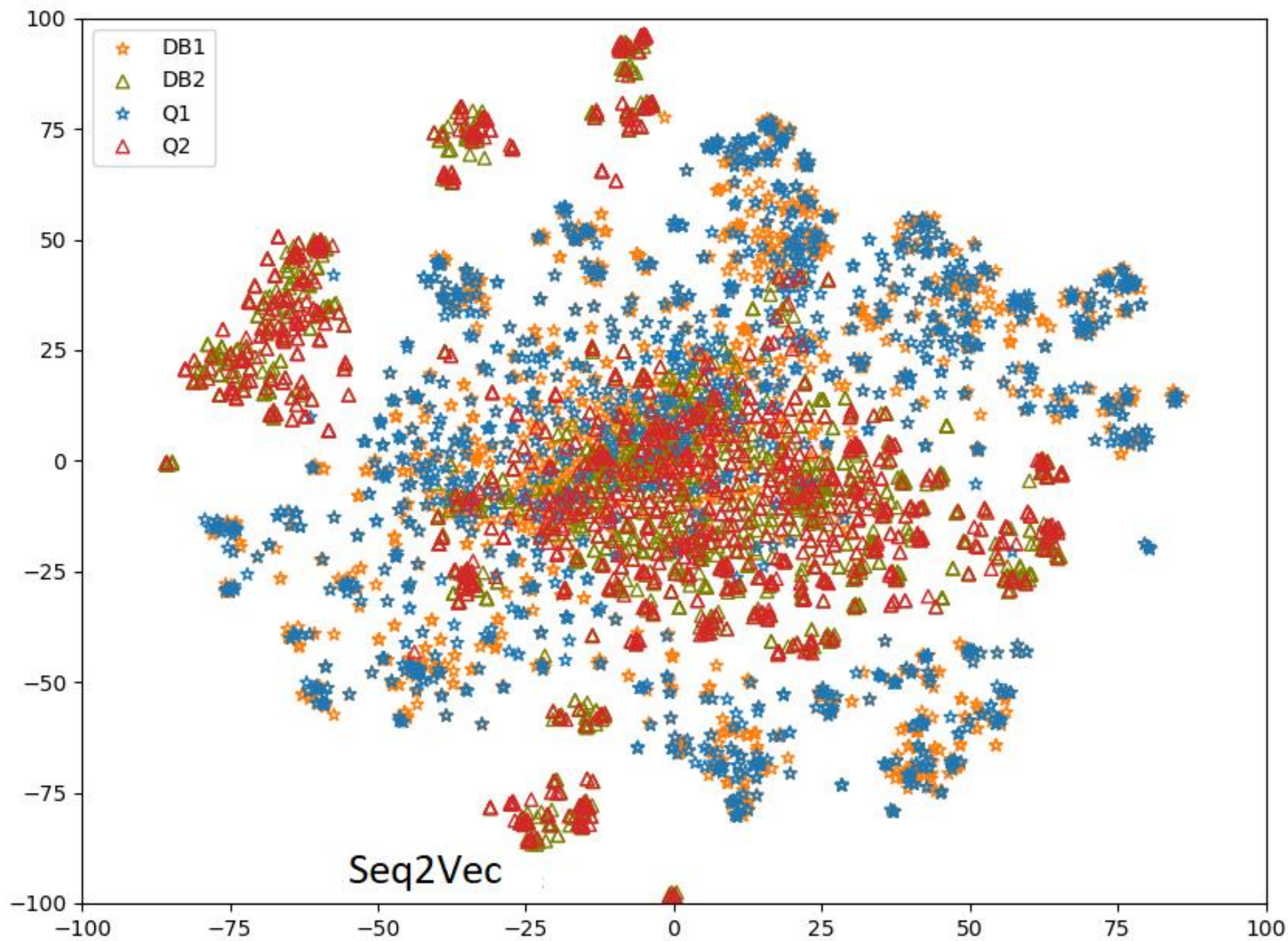

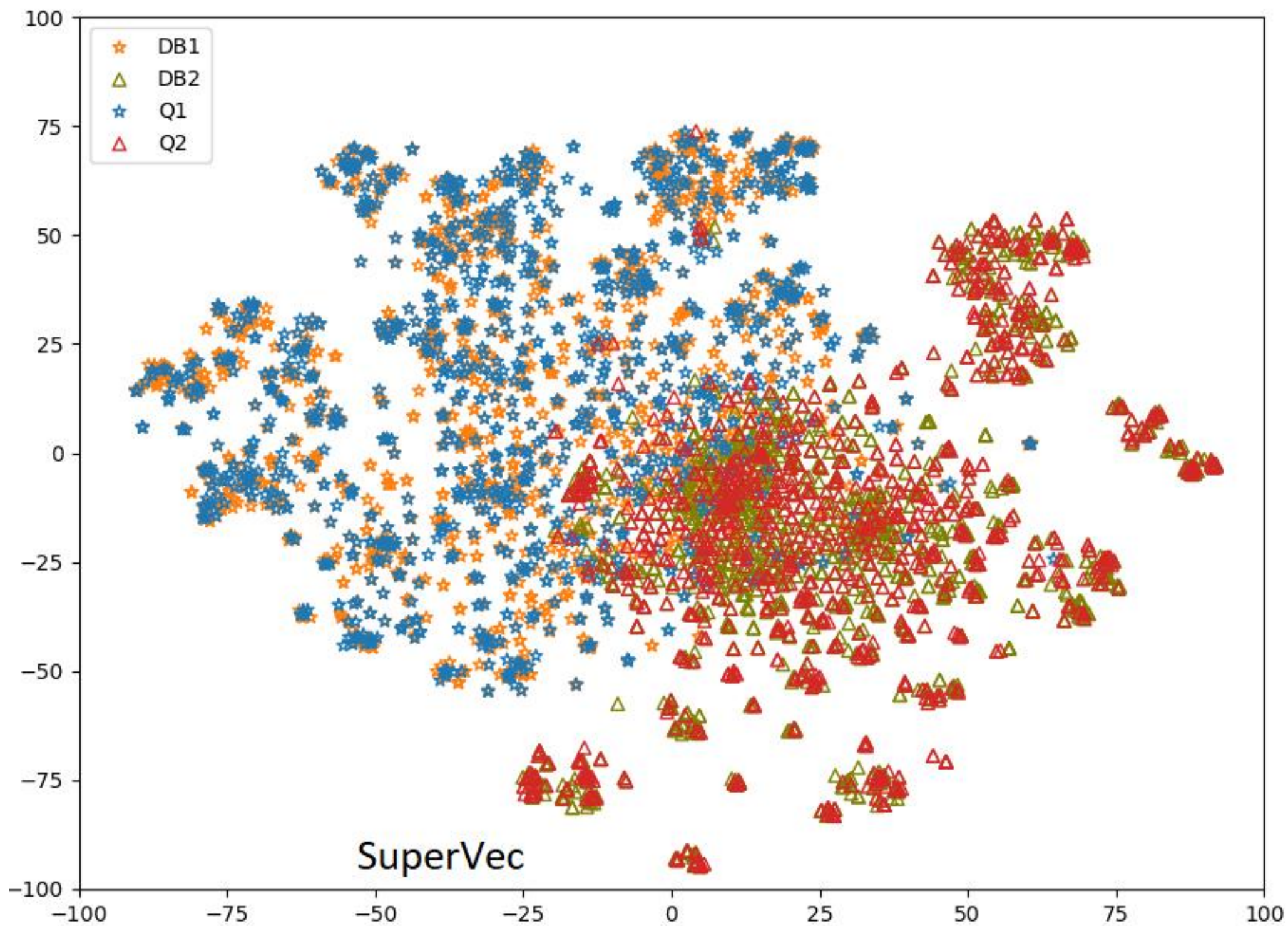

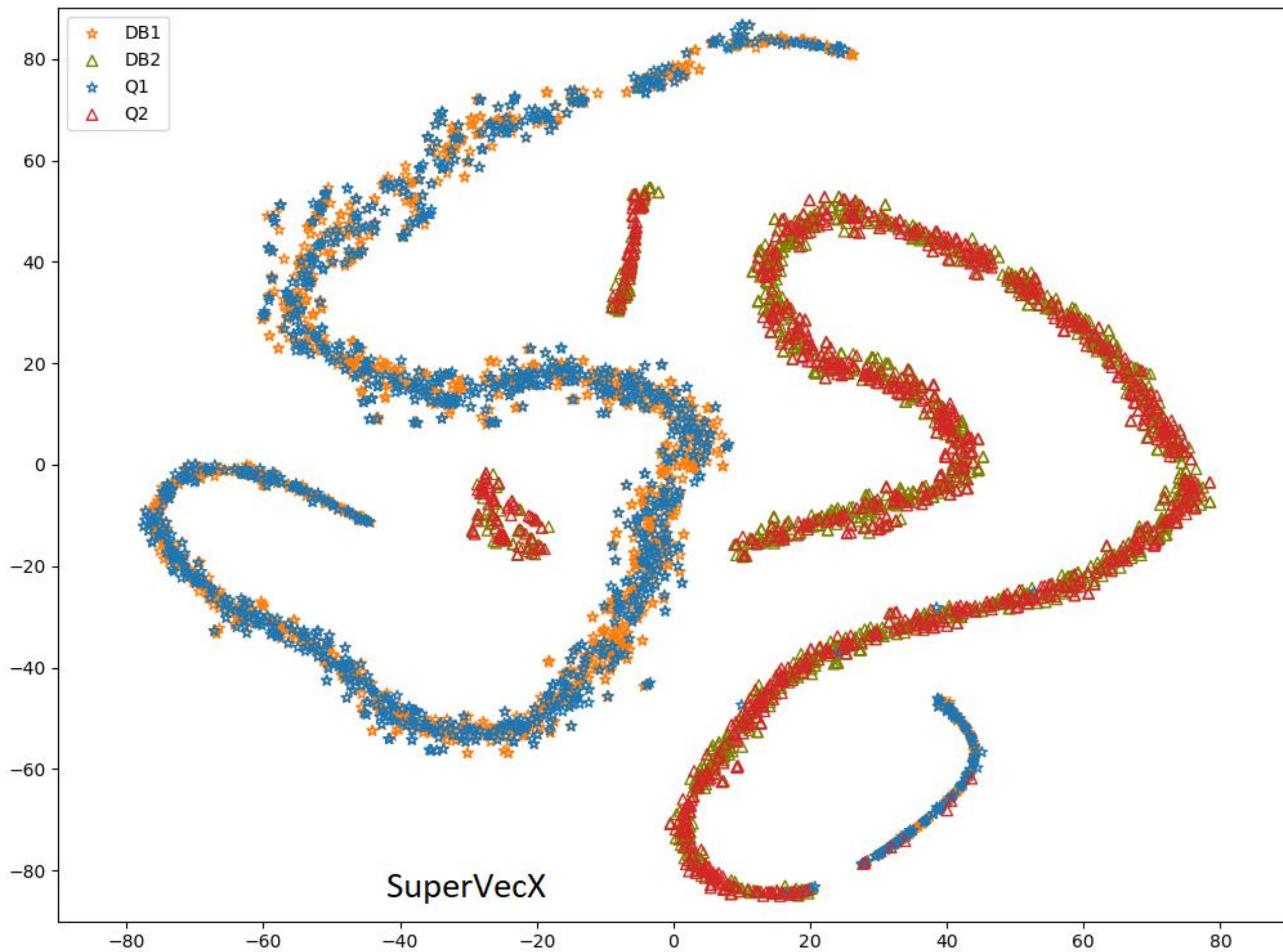

Supplement: S1 Fig — (PDF) [file pone.0216636.s005.pdf]
